# Ultrasound‐Responsive Piezoelectric Membrane Promotes Osteoporotic Bone Regeneration via the “Two‐Way Regulation” Bone Homeostasis Strategy

**DOI:** 10.1002/advs.202504293

**Published:** 2025-04-28

**Authors:** Xinhui Wu, Tianlong Wang, Jinhui Zhao, Lei Zhang, Zhiqing Liu, Yixing Chen, Yiping Luo, Yaqi Liu, Yan Chen, Hui Jiang, Dilixiati Duolikun, Junjian Liu, Wentao Cao, Longpo Zheng

**Affiliations:** ^1^ Department of Orthopedics Shanghai Tenth People's Hospital School of Medicine Tongji University Shanghai 200072 China; ^2^ Department of Prosthodontics Shanghai Stomatological Hospital & School of Stomatology Fudan University Shanghai 201102 China; ^3^ Shanghai Trauma Emergency Center Shanghai 200072 China; ^4^ Orthopedic Intelligent Minimally Invasive Diagnosis & Treatment Center Shanghai Tenth People's Hospital Tongji University School of Medicine Shanghai 200072 China

**Keywords:** bone homeostasis, osteoporotic bone repair, piezoelectric stimulation, two‐way regulation, ultrasound

## Abstract

The repair of osteoporotic bone defects remains inadequately addressed, primarily due to a disruption in bone homeostasis, characterized by insufficient bone formation and excessive bone resorption. Current research either focuses on promoting bone formation or inhibiting bone resorption, however, the bone repair efficacy of these single‐target therapeutic strategies is limited. Herein, a “two‐way regulation” bone homeostasis strategy is proposed utilizing piezoelectric composite membranes (DAT/KS), capable of simultaneously regulating osteogenesis and osteoclastogenesis, with high piezoelectric performance, good biocompatibility, and excellent degradability, to promote bone regeneration under osteoporotic conditions. The DAT/KS membrane under ultrasound (US) treatment enables the controlled modulation of piezoelectric stimulation and the release of saikosaponin D (SSD), which promotes osteogenic differentiation while simultaneously inhibiting osteoclast differentiation and function, thereby effectively restoring bone homeostasis and enhancing osteoporotic bone repair. Mechanistic insights reveal the promotion of both canonical and non‐canonical Wnt signaling in bone marrow mesenchymal stem cells (BMSCs), which determines their osteogenic differentiation fate, and the downregulation of the NF‐κB signaling in bone marrow mononuclear macrophages (BMMs). This study presents optimized sono‐piezoelectric biomaterials capable of bidirectionally regulating both osteogenic and osteoclastic differentiation, providing a new potential therapeutic approach for pathological bone injuries.

## Graphical abstract

Patients with osteoporosis face a higher risk of bone injury, mainly due to weakened osteogenic differentiation and bone formation, along with increased osteoclast differentiation and bone resorption activity, leading to bone homeostasis imbalance. A “two‐way regulation” strategy is proposed to simultaneously modulate both osteogenesis and osteoclastogenesis, aiming to address the challenges of bone repair under osteoporotic conditions.

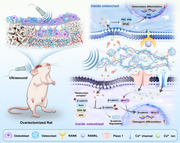

## 1 Introduction

With the aging of the global population, osteoporosis has become a common chronic disease, predominantly affecting the elderly and postmenopausal women.^[^ ^1^ ^]^ Bone tissue, as a highly dynamic organ, undergoes continuous remodeling through osteoblast‐mediated bone formation and osteoclast‐mediated bone resorption.^[^ ^2^ ^]^ When bone homeostasis is disrupted, increased osteoclast formation and excessive bone resorption lead to osteoporosis and other osteolytic diseases.^[^ ^3^ ^]^ The typical features of osteoporosis include disruption of bone microstructure, bone mass loss, and increased bone fragility, all of which increase the risk of fractures and bone defects. Due to reduced osteoblast‐mediated bone formation and excessive osteoclast‐mediated bone resorption, bone homeostasis is disrupted, which poses a major challenge in the clinical treatment of bone defects under osteoporotic conditions.^[^ ^4^ ^]^ Therefore, implant materials for treating osteoporotic bone defects should effectively regulate bone homeostasis, promote osteogenesis, inhibit osteoclast activity, and ultimately facilitate bone defect repair.

Electrical signals regulate the physiological functions of the human body.^[^ ^5^ ^]^ Exogenous electrical stimulation or restoration of the electrical microenvironment holds significant promise in the field of tissue defect repair.^[^ ^6^ ^]^ The endogenous electric field in bone regulates cellular processes such as growth, proliferation, differentiation, and migration.^[^ ^7^ ^]^ Many studies showed that mechanical force applied to piezoelectric biomaterials generates surface charges, and placing them at bone defect sites can accelerate the bone repair process.^[^ ^8^ ^]^ Compared to normal bone tissue, bone defect repairing requires a stronger piezoelectric potential, and mechanical pressure alone is insufficient to generate enough piezoelectric charge.^[^ ^9^ ^]^ Medical US therapy can focus sound waves on specific lesion sites in the body to achieve therapeutic effects such as ablation, pain relief, and promotion of blood circulation.^[^ ^10^ ^]^ US waves apply mechanical vibrations to piezoelectric biomaterials, converting mechanical vibrations into piezoelectric potentials. By utilizing the remote programming, adjustable intensity, and spatiotemporal specificity of US treatment, and combining it with piezoelectric biomaterials, sufficient electrical signal output can be generated at bone defect sites, thereby facilitating accelerated bone tissue regeneration.^[^ ^11^, ^13^ ^]^ However, the non‐degradability of most piezoelectric implants necessitates secondary surgeries, which somewhat limits their clinical application value. The development of high‐performance piezoelectric biomaterials with good biocompatibility and degradability and has emerged as a critical requirement in regenerative medicine.

Electrical stimulation enhances cellular energy metabolism, accelerates the recruitment of BMSCs to the bone repair area, and activates mechanosensitive ion channel component 1 (Piezo1) and voltage‐gated channels.^[^ ^12^ ^]^ These biological effects upregulate osteogenesis‐related proteins and multiple signaling pathways, including Wnt signaling, Phosphoinositide 3‐kinase/Protein kinase B (PI3K/AKT) signaling, and Mitogen‐activated protein kinase (MAPK) signaling, thereby determining their osteogenic differentiation fate.^[^ ^13^ ^]^ Wnt signaling, as a crucial pathway regulating osteoblast differentiation, plays an important role in bone development and bone homeostasis.^[^ ^14^ ^]^ The Wnt signaling pathway is classified into two primary branches: canonical and non‐canonical. The canonical Wnt signaling is primarily mediated by *β*‐catenin. In the absence of Wnt activation, cytoplasmic *β*‐catenin is phosphorylated by a destruction complex consisting of glycogen synthase kinase‐3*β* (GSK‐3*β*), Adenomatous polyposis coli (APC), and Axis Inhibition Protein 2 (Axin2). Wnt activation inhibits GSK‐3*β* activity and promotes the cytoplasmic accumulation of *β*‐catenin. The accumulated *β*‐catenin translocates to the nucleus, where it activates the expression of target genes.^[^ ^15^ ^]^ The activation of Wnt/*β*‐catenin signaling inhibits mesenchymal stem cell (MSC) commitment to the chondrogenic and adipogenic lineages while promoting commitment to and differentiation along the osteoblastic lineage.^[^ ^16^ ^]^ The non‐canonical signaling refers to pathways that are not mediated by *β*‐catenin. Ligands such as Wnt5a and Wnt11 activate the Wnt/Ca^2+^ and Wnt/PCP pathways without inducing intracellular *β*‐catenin accumulation.^[^ ^17^ ^]^ In the Wnt/Ca^2+^ pathway, an increase in intracellular Ca^2+^ concentration activates calmodulin‐dependent protein kinase II (CaMK II) and protein kinase C (PKC). This subsequently activates the nuclear factor of activated T cells (NFAT) family of transcription factors, which are involved in various cellular processes, including cell adhesion, migration, and differentiation.^[^ ^18^ ^]^ Researches have shown that activation of the Piezo1 and voltage‐gated calcium channels on the BMSC membrane triggers Ca^2+^ influx, which subsequently activates the NFAT family, accelerating the osteogenic differentiation process.^[^ ^19^ ^]^

**Figure 1:**
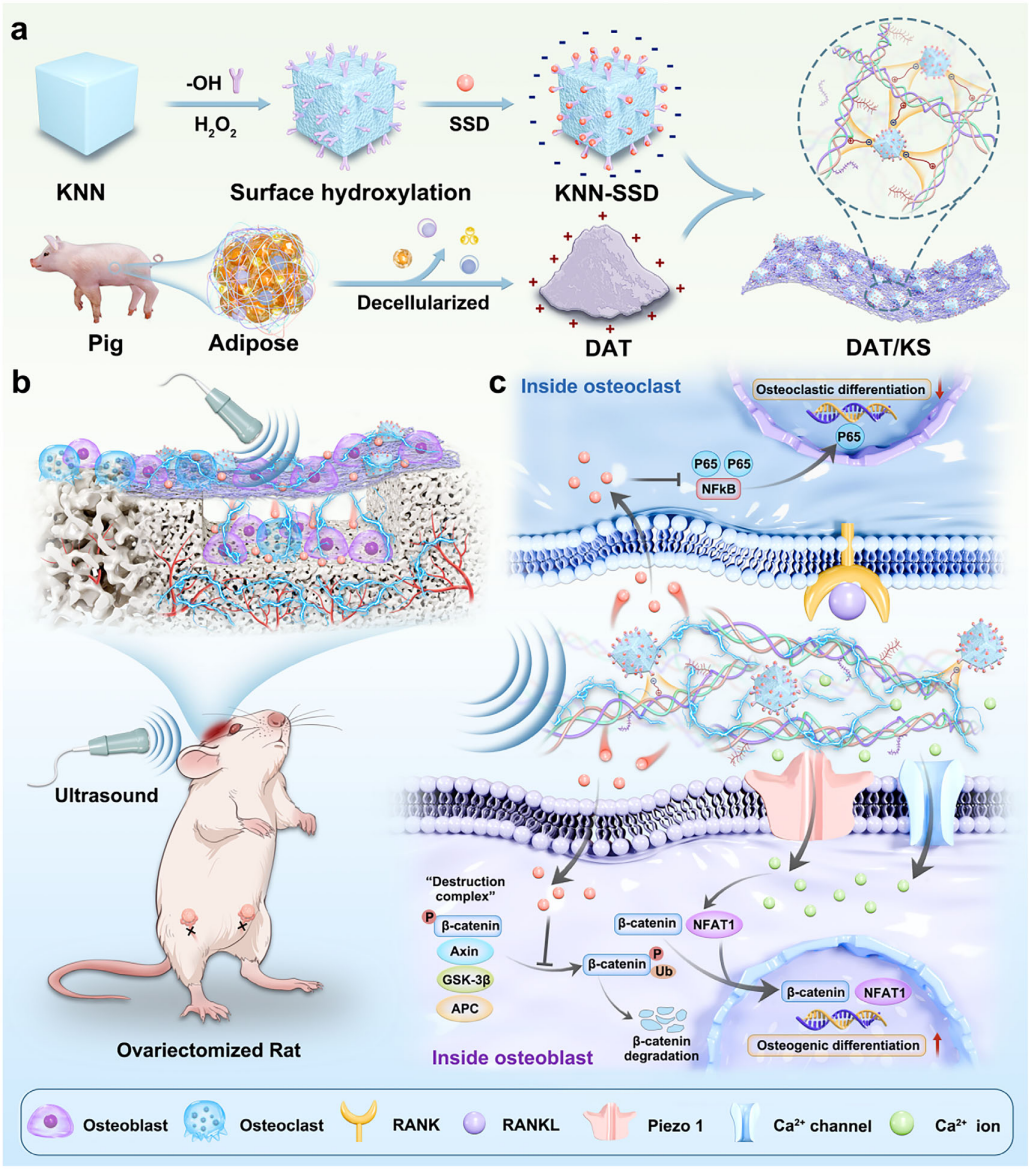
Schematic illustration of US‐driven the piezoelectric membrane implementing the “two‐way regulation” bone homeostasis strategy to accelerate osteoporotic bone defect repair a) Preparation of the DAT/KS membrane. b) The combined effects of piezoelectric stimulation and SSD release promote osteogenesis and angiogenesis, while inhibiting osteoclast differentiation, thereby accelerating bone defect repair in an ovariectomized rat. c) Potential mechanism of US‐driven the DAT/KS membrane in bidirectional regulation of osteogenic and osteoclastic differentiation.

Simultaneously achieving bidirectional regulation of osteogenic and osteoclastic differentiation to restore bone homeostasis is crucial for accelerating bone healing under osteoporotic conditions. However, relying solely on electrical stimulation to promote osteogenic differentiation and new bone formation, while neglecting the consumption of newly formed bone matrix by osteoclasts, is insufficient for effective bone repair. Our previous research found that the traditional Chinese medicine extract SSD effectively inhibits osteoclast differentiation and function, alleviating osteoclast‐mediated bone loss in vivo, without toxic side effects.^[^ ^20^ ^]^ In this study, we modified the hydroxylated lead‐free piezoelectric material K_0.5_Na_0.5_NbO_3_ (KNN) with the NF‐κB signaling inhibitor SSD to synthesize KNN‐SSD nanoparticles. The obtained nanoparticles were then incorporated into decellularized adipose tissue (DAT) by vacuum filtration to prepare a piezoelectric composite membrane (DAT/KS) with good biocompatibility, satisfactory mechanical strength, and excellent degradability (**Figure** **1**a). The US‐driven DAT/KS membrane enables the controllable modulation of electrical stimulation and SSD release, enhancing cellular energy status and accelerating BMSC recruitment. The DAT/KS membrane achieves the “two‐way regulation” of osteogenic and osteoclastic differentiation, promoting bone formation while inhibiting excessive bone resorption, thus facilitating bone defect healing under osteoporotic conditions. Mechanistically, upregulation of both canonical and non‐canonical Wnt signaling in BMSCs governs osteogenic differentiation fate, while NF‐κB signaling inhibition in BMMs weakens osteoclast differentiation and bone resorption (Figure 1b,c). This study successfully achieved bidirectional regulation of osteogenesis and osteoclastogenesis under osteoporotic conditions, providing a promising innovative strategy to address the challenges of osteoporotic bone repair in clinical settings.

## 2 Results and Discussion

### 2.1 Preparation and Characterization of the DAT/KS Membrane

**Figure 2:**
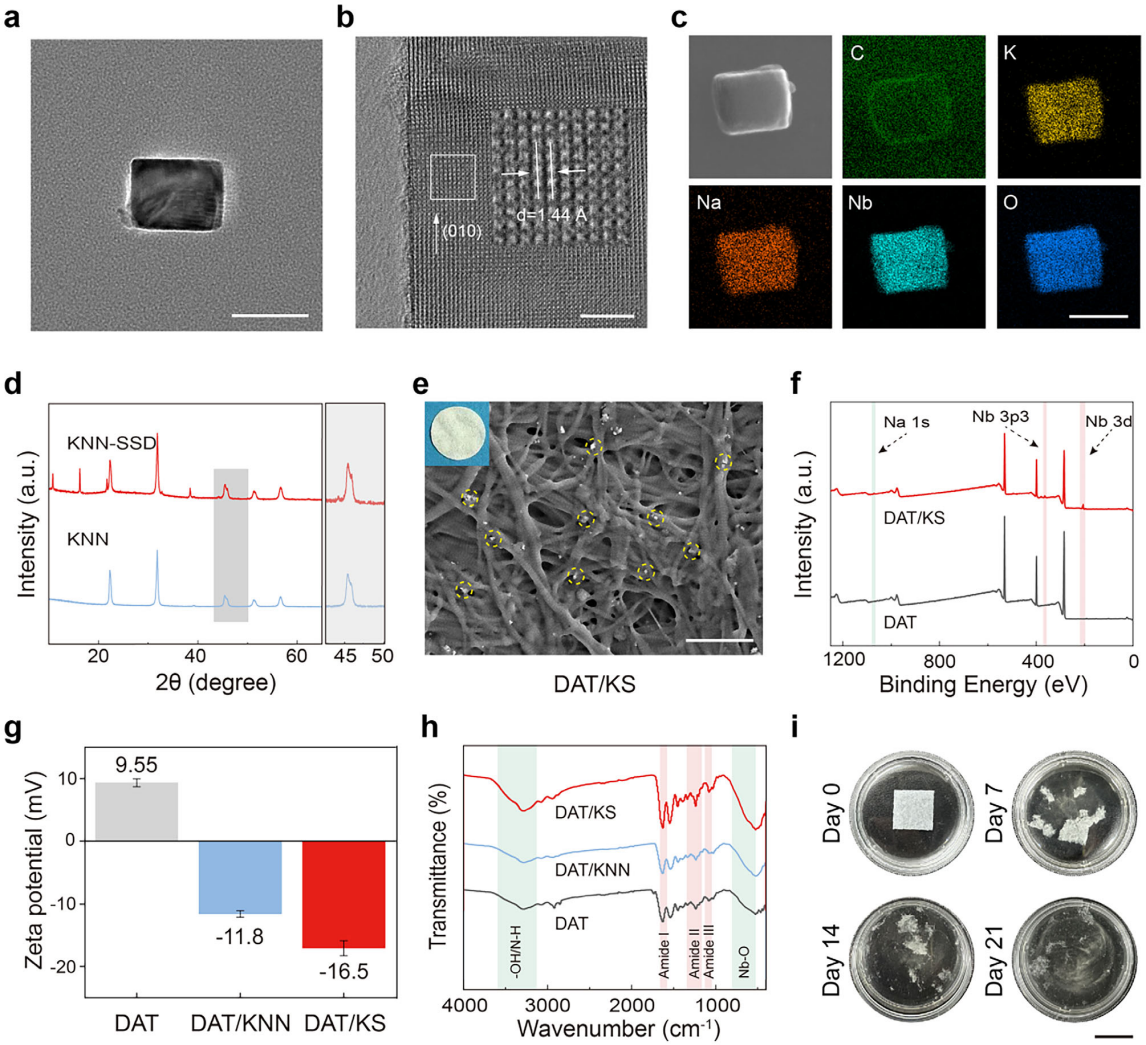
Preparation and characterization of the DAT/KNN and DAT/KS membranes. a,b) Bright‐field TEM and HRTEM images of KNN nanoparticles. c) EDS elemental mapping of KNN‐SSD nanoparticles. d) XRD patterns of KNN and KNN‐SSD nanoparticles. e) The Digital photograph (top left) and SEM image show the morphology of the DAT/KS membrane. The yellow dashed circles indicate KNN‐SSD nanoparticles attached to the DAT fibers. f) XPS spectra of the DAT and DAT/KS membranes. g) Zeta potential measurements of the DAT, DAT/KNN, and DAT/KS membranes. h) FTIR spectra of the DAT, DAT/KNN, and DAT/KS membranes. i) Representative images of the DAT/KS membrane degradation on days 0, 7, 14, and 21 in PBS with collagenase. Scale bars, 50 nm (a,c), 5 nm (b), 1 µm (e), 2 cm (i).

The obtained KNN piezoelectric nanoparticles after calcination were first analyzed. The morphology of the aggregated KNN nanoparticles is shown in Figure S1a (Supporting Information), observed under transmission electron microscopy (TEM). Energy‐dispersive X‐ray spectroscopy (EDX) elemental mapping analysis revealed the distribution of constituent elements. The atomic ratio of K/Na is ≈1 (Figure S1b, Supporting Information). The morphology of individual KNN nanoparticle is presented as block‐like structures under TEM (**Figure** **2**a). Selected area electron diffraction (SAED) patterns reveal the highly ordered crystalline structure of KNN nanoparticle (Figure S2, Supporting Information). High‐resolution transmission electron microscopy (HRTEM) images further confirmed the (010) crystal planes of KNN nanoparticle by measuring the interplanar spacing (1.44 Å, Figure 2b). The TEM image and EDS elemental mapping of KNN‐SSD nanoparticle showed that the four main elements, K, Na, Nb, and O, are uniformly distributed. Furthermore, the uniform distribution of C on the surface of KNN further confirmed the surface modification by SSD (Figure 2c). X‐ray diffraction (XRD) analysis indicated that both KNN and KNN‐SSD exhibit a perovskite ABO₃ crystal structure, with a split diffraction peak observed at 2θ = 46°, which is associated with a tetragonal phase (Figure 2d).^[^ ^13b^ ^]^ According to Fourier Transform Infrared (FTIR) spectroscopy results, KNN‐SSD not only exhibits the characteristic peak corresponding to the stretching vibration of the Nb‐O bond (≈650 cm^−^¹), but also shows other notable absorption peaks in different wavenumber ranges.^[^ ^21^ ^]^ The broad absorption peak at 3200–3600 cm^−^¹ is attributed to the stretching vibration of ─OH groups, indicating that the surface of KNN nanoparticles is modified by SSD. The characteristic peaks at 2800–3000 cm^−^¹ correspond to the asymmetric and symmetric stretching vibrations of C‐H (alkyl), while the peaks at 1000–1300 cm^−¹^ are associated with C‐O bond vibrations (Figure S3, Supporting Information). Dynamic light scattering (DLS) analysis revealed that there is no significant difference in particle size between KNN and KNN‐SSD nanoparticles. Due to aggregation, the particle size is ≈286.1 nm, which is larger than that of individual nanoparticle observed in the TEM image (Figure S4, Supporting Information).

In recent years, natural extracellular matrix (ECM) has garnered significant attention in the field of bone repair.^[^ ^22^ ^]^ As a form of decellularized extracellular matrix (dECM), DAT has emerged as a viable option for repairing bone defects due to its ease of availability.^[^ ^23^ ^]^ DAT consists of numerous fibrous strands, and SEM reveals a porous structure, with the top‐left image showing a digital photograph (Figure S5, Supporting Information). Based on Oil Red O, HE, Masson's Trichrome, and DAPI staining results, the DAT samples successfully removed lipids, cells, and DNA while retaining the extracellular matrix, especially collagen, compared to adipose tissue (Figure S6a, Supporting Information). Quantitative analysis of DNA content indicates that DAT samples nearly completely removed cells and DNA (Figure S6b, Supporting Information). Figure S7 (Supporting Information) shows the changes in the general morphology of DAT/KS membranes with different KNN‐SSD weight ratios (w/w, 10%, 20%, and 30%). As the KNN‐SSD weight ratio increased, the color of the DAT/KS membranes transitions from translucent to white. The digital photograph (top‐left) and SEM image of the DAT/KS membrane are shown in Figure 2e, where the yellow dashed circles (representative) highlight the white KNN‐SSD nanoparticles attached to the fibrous strands of DAT. The DAT/KS membrane preserves its structural integrity both in the dry state and when exposed to water at room temperature, demonstrating excellent flexibility suitable for applications in liquid environments (Figure S8a, Supporting Information). The thickness of the DAT/KS membrane was measured to be 0.126 mm using a thickness gauge (Figure S8b, Supporting Information). The surface average roughness (Ra) of the DAT/KS membrane was observed using Atomic Force Microscopy (AFM), with a Ra value of 265 nm (Figure S8c, Supporting Information). The water contact angles of the DAT, DAT/KNN, and DAT/KS membranes are shown in Figure S9a (Supporting Information), indicating good hydrophilicity for all three membranes, with no significant difference in water contact angle (Figure S9b, Supporting Information). Due to the modification of KNN‐SSD nanoparticles, XPS spectra confirmed the presence of Nb and Na elements in the DAT/KS membrane (Figure 2f). The Zeta Potential results indicated that the DAT samples exhibit a positive zeta potential, whereas the synthesized DAT/KS samples show a negative charge (Figure 2g). In the FTIR spectrum, the DAT/KS membrane exhibited amide bands (Amide I, II, III) derived from DAT, along with a characteristic Nb‐O absorption peak (≈650 cm^−^¹) and a broad absorption peak corresponding to ‐OH/N‐H stretching vibrations (3200–3600 cm^−^¹).^[^ ^22^ ^]^ These results suggest that the DAT/KS membrane successfully incorporates Nb‐O groups while retaining the key protein structural features and surface functional groups of DAT (Figure 2h). The tensile stress‐strain curve indicated that the DAT membrane possesses certain tensile strength and ductility. The incorporation of KNN and KNN‐SSD nanoparticles into the DAT samples enhances the tensile strength of the membranes (Figure S10, Supporting Information). UV–vis analysis revealed a good linear relationship between SSD concentration and absorbance (Figure S11a, Supporting Information), while ultrasonic treatment significantly accelerated the release rate of SSD from the DAT/KS membrane, particularly during the early release phase, indicating that ultrasonic treatment enables more efficient drug release (Figure S11b, Supporting Information). Additionally, we assessed the degradation performance of the DAT/KS membrane. In a PBS solution containing 5 CDU mL^−1^ collagenase, The DAT/KS membrane was fragmented by Day 7 and underwent nearly complete degradation by Day 21 (Figure 2i), with the remaining weight of the membrane at different time points shown in Figure S12 (Supporting Information).

### 2.2 Piezoelectric Properties of the DAT/KS Membrane

**Figure 3:**
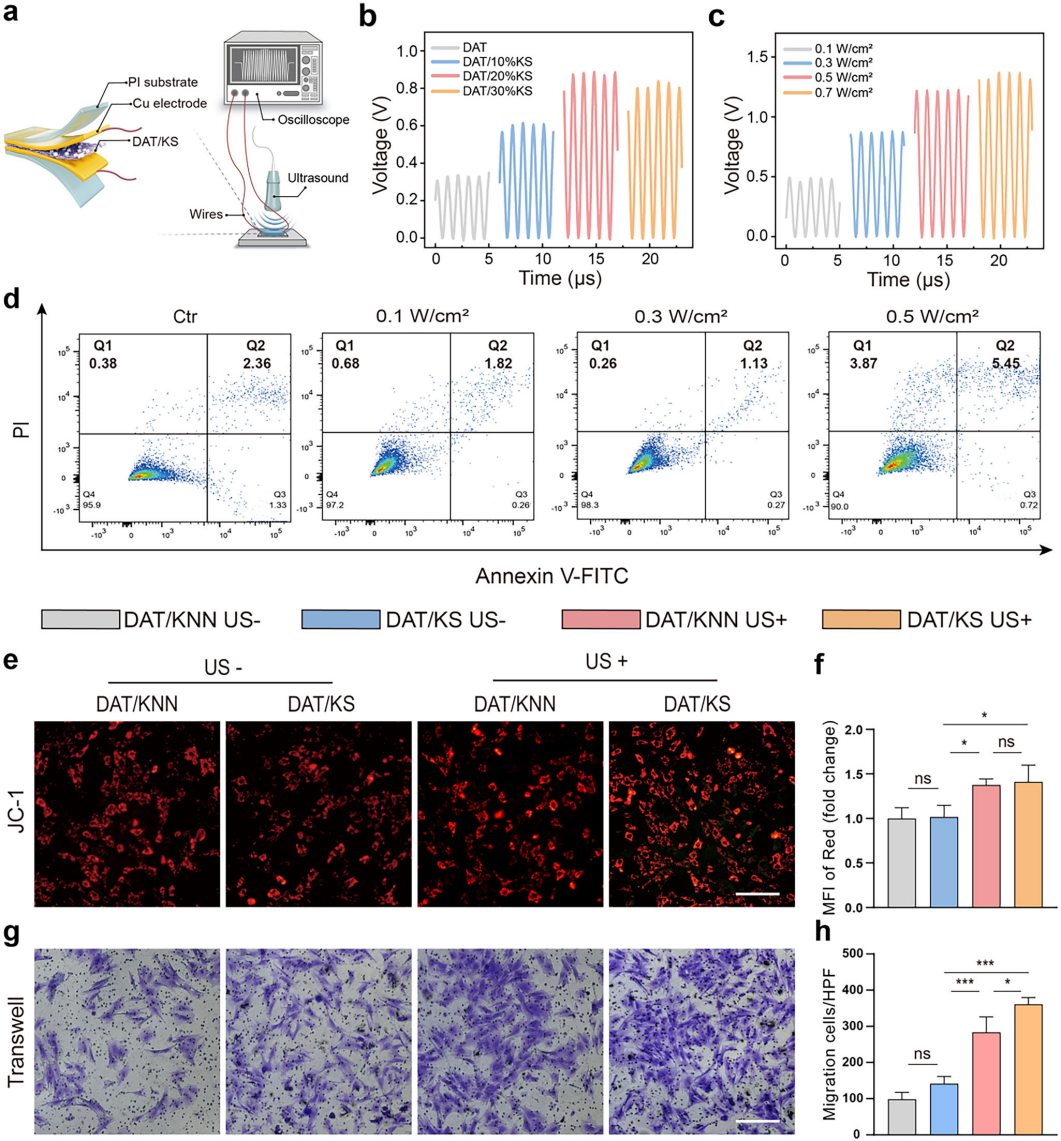
The piezoelectric properties of the membranes and their effects on the proliferation and energy status of BMSCs. a) Schematic illustration of the assembly of the DAT/KS membrane sandwich structure and its electrical output measurement. b) Voltage output of the DAT/KS membrane with varying KNN‐SSD mass ratios (10%, 20%, and 30%) under uniform US stimulation. c) Voltage output of the DAT/KS membranes under different US intensities (0.1, 0.3, and 0.5 W cm^−^^2^). d) Flow cytometry analysis of BMSCs co‐cultured with the DAT/KS membrane under different US intensities (0.1, 0.3, 0.5 W cm^−^^2^). e) JC‐1 staining of BMSCs co‐cultured with the DAT/KNN and DAT/KS membranes under conditions with and without US treatment. f) Semi‐quantitative analysis of mitochondrial membrane potential via MFI of JC‐1 red aggregates (n  =  3, mean ± s.d.). g) Transwell migration assay of BMSCs co‐cultured with the DAT/KNN and DAT/KS membranes under conditions with and without US treatment. h) Semi‐quantitative analysis of migrated cells per high‐power field (HPF) (n  =  3, mean ± s.d.). n represents the number of biologically independent samples. Scale bar, 400 µm (e, g). (*P < 0.05, ***P < 0.001).

The piezoelectric constants *d*_33_ of the DAT/KS membranes were directly measured to assess their piezoelectric performance. To ensure the accuracy and reliability of the measurements, the *d*_33_ values were determined at three random locations on each DAT/KS membrane and then averaged. The results indicated that the *d*_33_ values initially increased with the addition of KNN‐SSD nanoparticles, reaching a maximum value of 13.6 pC/N at KNN‐SSD loading of 20% (Figure S13, Supporting Information). However, further increases in the KNN‐SSD content led to a decrease in the *d*_33_ values. This trend can be attributed to the aggregation of excessive KNN‐SSD nanoparticles within the DAT/KS membrane, which introduces interfacial defects between the DAT and KNN‐SSD phases. These defects disrupt the effective stress transfer and reduce the overall piezoelectric performance of the composite. To further evaluate the piezoelectric performance of the DAT/KS membranes under dynamic conditions, thin‐film nanogenerators were fabricated, comprising the DAT/KS membranes, Cu electrodes, and polyimide (PI) substrate layers (**Figure** **3**a). The nanogenerators were subjected to US stimulation at an intensity of 0.3 W cm^−^^2^. As shown in Figure 3b, the peak‐to‐peak output voltage (Vpp) of the DAT/KS nanogenerators initially increased from 0.34 V to 0.89 V with increasing KNN‐SSD mass ratio (0 to 20%) but subsequently decreased to 0.83 V at a KNN‐SSD mass ratio of 30%. This behavior is consistent with the observed trend in the *d*_33_ values, further confirming the detrimental effects of excessive KNN‐SSD on the membrane's piezoelectric properties. Additionally, the output piezoelectric signals of the DAT/KS nanogenerators were systematically characterized under various US intensities ranging from 0.1 to 0.7 W cm^−^^2^. Notably, the electric outputs of the nanogenerators were found to be proportional to the applied US intensity. Specifically, the output voltage of the DAT/KS nanogenerators gradually increased from 0.49 to 1.37 V as the US intensity was increased from 0.1 to 0.7 W cm^−^^2^ (Figure 3c). For ultrasound intensities of 0.1, 0.3, 0.5, and 0.7 W cm^−^^2^, the calculated radiation forces are 0.8, 2.4, 4.0, and 5.6 mN, respectively. This linear relationship between the output voltage and US intensity highlights the potential of the DAT/KS nanogenerators for electrotherapy applications under different US stimulation conditions.

### 2.3 US‐Driven the DAT/KS Membrane Promotes Cell Proliferation and Energy States

As shown in Figure S14 (Supporting Information), the schematic illustrates the co‐culture of BMSCs with the piezoelectric composite membrane in a 6‐well plate, followed by US treatment. The sterilized piezoelectric composite membranes were uniformly placed in the wells of the plate. The US probe was positioned at the bottom, enabling controlled electrical stimulation and SSD release. SEM images showed that BMSCs grew well on the piezoelectric composite membrane, with their pseudopodia being fully extended and strongly adhered to the porous surface of the membrane (Figure S15a, Supporting Information). Live/Dead staining results further confirmed that the DAT/KS and DAT/KNN membranes exhibited no significant toxicity and demonstrated good biocompatibility with BMSCs (Figure S15b, Supporting Information). BMSCs were co‐cultured with the DAT/KS membrane, and treated with different ultrasonic intensities (0.1 W cm^−^^2^, 0.3 W cm^−^^2^, 0.5 W cm^−^^2^) daily. After 48 h, apoptosis assays were conducted on each experimental group. Flow cytometry analysis showed that at an US intensity of 0.3 W cm^−^^2^, the mechanical damage (0.26%) and apoptosis (1.13%) were the lowest compared to other groups. However, at an US power of 0.5 W cm^−^^2^, mechanical damage and apoptosis in BMSCs reached 3.87% and 5.45%, respectively (Figure 3d). Therefore, an US intensity of 0.3 W cm^−^^2^ was selected for subsequent experiments. Previous studies have demonstrated that piezoelectric stimulation promotes cell proliferation, enhances cellular energy states, and facilitates cell adhesion and migration.^[^ ^24^ ^]^ Figure S16 (Supporting Information) indicates that BMSCs in all experimental groups grew well, with no significant dead cells observed. A higher proportion of cells were present in the DAT/KNN + US and DAT/KS + US groups. The CCK‐8 assay results were consistent, showing significantly enhanced BMSC proliferation in the DAT/KNN + US and DAT/KS + US groups compared to the Ctr (control) group on days 3 and 5 (Figure S17, Supporting Information). Mitochondrial membrane potential is commonly used to indicate the cellular oxidative metabolic state.^[^ ^25^ ^]^ We assessed the mitochondrial membrane potential of BMSCs. In the DAT/KNN + US and DAT/KS + US groups, the mean fluorescence intensity (MFI) of red fluorescence detected by the JC‐1 probe were significantly increased. This fluorescence enhancement indicates that the cellular energy state was improved (Figure 3e,f). Transwell assay results showed that cell migration was significantly enhanced in the DAT/KNN + US and DAT/KS + US groups (Figure 3g,h). Similarly, the wound healing assay indicated that BMSCs migrated a longer distance in the US treated groups (Figure S18, Supporting Information). These findings suggest that electrical stimulation promotes BMSC migration and enhances cell recruitment, thereby optimizing the osteogenic microenvironment.

### 2.4 US‐Driven the DAT/KS Membrane Accelerates Osteogenic Differentiation

**Figure 4:**
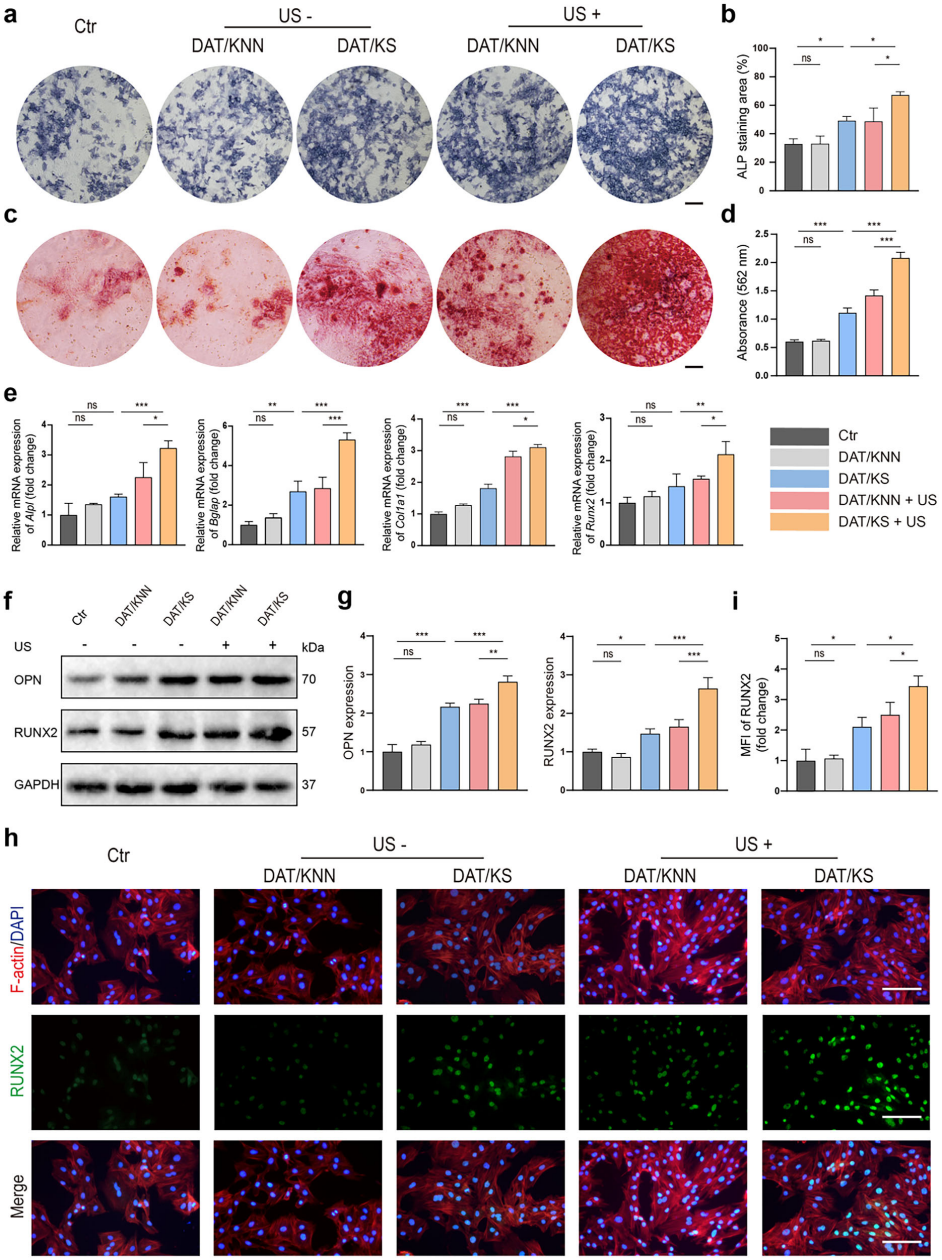
The effects of piezoelectric composite membranes on osteogenic differentiation. a) ALP staining of BMSCs on day 7 of osteogenic differentiation in different experimental groups: Ctr, DAT/KNN, DAT/KS, DAT/KNN + US, and DAT/KS + US. b) Semi‐quantitative analysis of ALP positive area (n  =  3, mean ± s.d.). c) ARS staining on day 21 of osteogenic differentiation to evaluate mineralization of BMSCs in different experimental groups. d) Quantitative analysis of absorbance at 562 nm (n  =  3, mean ± s.d.). e) Relative mRNA expression levels of osteogenic markers (*Alpl*, *Bglap*, *Col1a1*, and *Runx2*) in BMSCs under different treatments. f) Western blot analysis of OPN and RUNX2 expression in BMSCs. g) Semi‐quantitative analysis of OPN and RUNX2 protein expression levels from Western blot results (n  =  3, mean ± s.d.). h) Immunofluorescence images showing F‐actin (red) and RUNX2 (green) in BMSCs, with DAPI (blue) for nuclear staining. i) Semi‐quantitative analysis of RUNX2 MFI in different experimental groups (n  =  3, mean ± s.d.). n represents the number of biologically independent samples. Scale bar, 200 µm (a,c,h). (*P < 0.05, **P < 0.01, *** *P* < 0.001).

Alkaline phosphatase (ALP) is an enzyme highly expressed in osteoblasts, commonly used as an early marker of osteogenic differentiation and involved in the mineralization process of bone tissue.^[^ ^26^ ^]^ BMSCs were co‐cultured with the piezoelectric composite membranes and induced for osteogenic differentiation. The US treatment groups were subjected to daily US treatment. ALP staining was performed on day 7. The DAT/KS, DAT/KNN + US, and DAT/KS + US groups all promoted ALP expression and accelerated osteogenic differentiation (**Figure** **4**a). Among these, ALP expression was more pronounced in the DAT/KS + US group, indicating that piezoelectric stimulation and accelerated SSD release under US treatment facilitated osteogenic differentiation (Figure 4b). Consistently, on day 21 of osteogenic differentiation, Alizarin Red S (ARS) staining was used to assess the mineralization levels of osteoblasts. The US treated groups significantly enhanced the formation of mineralized nodules, with the DAT/KS + US group exhibiting the most prominent red staining (Figure 4c). ARS dye was eluted using cetylpyridinium chloride solution, and the absorbance of the eluate was measured at 562 nm to quantify the mineralization levels in each group. The DAT/KS + US group showed the highest level of osteoblast mineralization, accelerating osteogenic differentiation (Figure 4d). At the molecular level, the transcriptional levels of osteogenic markers, including *Alpl*, *Bglap*, *Col1a1*, and *Runx2*, were markedly increased in the DAT/KS + US group (Figure 4e). Likewise, the expression levels of osteogenic differentiation related proteins including OPN and RUNX2, were significantly upregulated in the DAT/KS + US group (Figure 4f,g). Furthermore, immunofluorescence staining was conducted to evaluate RUNX2 protein expression. Consistent with the aforementioned results, MFI of RUNX2 in the DAT/KS + US group was significantly higher than that in the other groups (Figure 4h,i).

### 2.5 Promotion of the Canonical and Non‐Canonical Wnt Signaling

**Figure 5:**
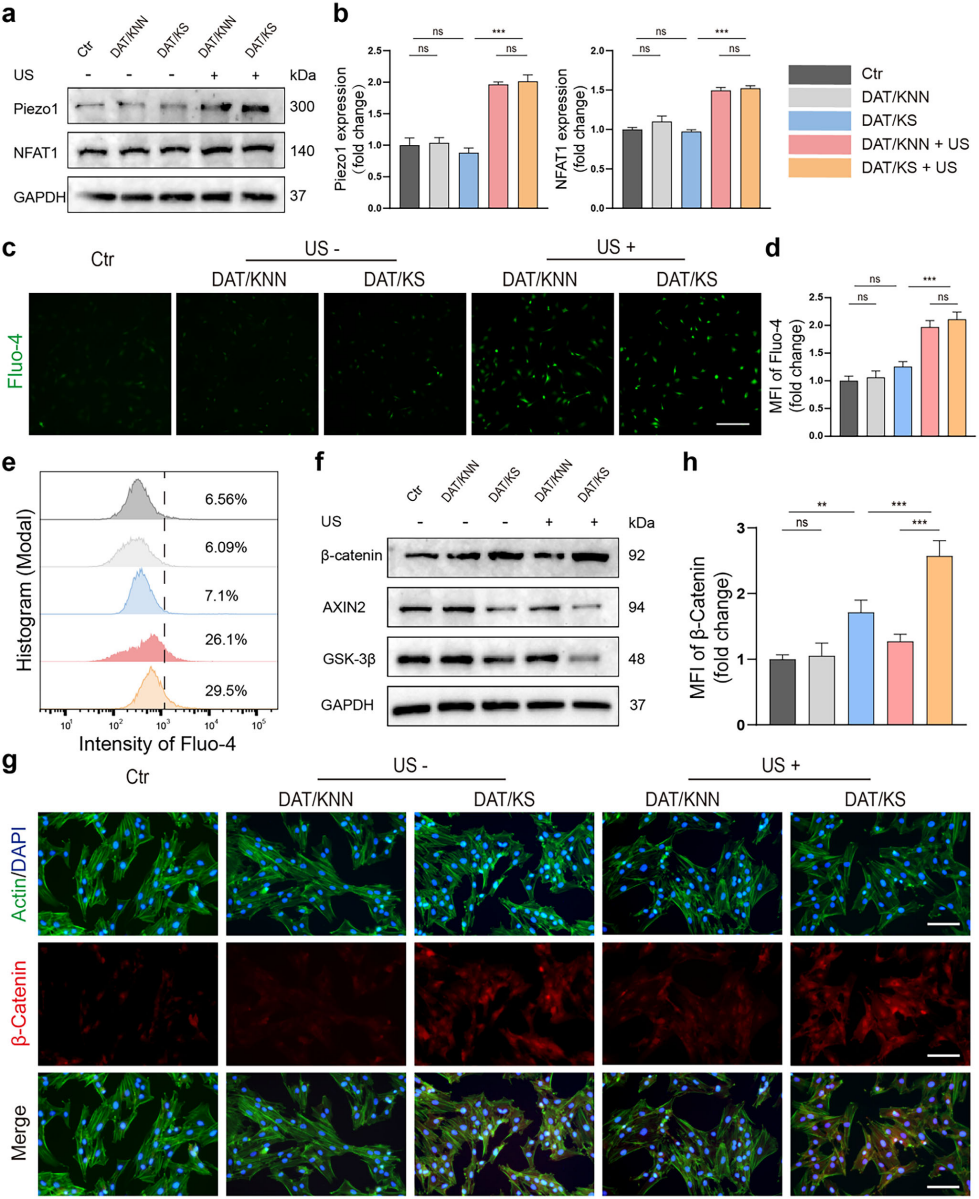
Regulation of canonical and non‐canonical Wnt signaling in BMSCs. a) Western blot analysis of Piezo1 and NFAT1 protein expressions in BMSCs from different experimental groups: Ctr, DAT/KNN, DAT/KS, DAT/KNN + US, and DAT/KS + US. b) Semi‐quantitative analysis of Piezo1 and NFAT1 protein expression levels from Western blot results (n  =  3, mean ± s.d.). c) Immunofluorescence staining of intracellular calcium levels using Fluo‐4 fluorescence probe in different experimental groups. d) Semi‐quantitative analysis of Fluo‐4 MFI (n  =  3, mean ± s.d.). e) Flow cytometry histogram of Fluo‐4 intensity showing intracellular calcium levels in different experimental groups, with percentages representing the proportion of positive cells. f) Western blot analysis of *β*‐catenin, AXIN2, and GSK‐3*β* protein expressions in BMSCs. g) Immunofluorescence staining showing *β*‐catenin (red), actin (green), and DAPI (blue) in different experimental groups. h) Semi‐quantitative analysis of *β*‐catenin MFI (n  =  3, mean ± s.d.). n represents the number of biologically independent samples. Scale bar, 400 µm (c), 200 µm (g). (**P < 0.01, *** *P* < 0.001).

The Wnt signaling serves as a crucial regulator of osteogenic differentiation and bone formation.^[^ ^14^ ^]^ When the Wnt/Ca^2^⁺ pathway is activated, calcium ions are mobilized from extracellular sources and the endoplasmic reticulum into the cytoplasm, subsequently triggering the activation of downstream effectors, including CaMKII (Calcium/Calmodulin‐dependent Protein Kinase II) and the NFAT (Nuclear Factor of Activated T‐cells) family.^[^ ^27^ ^]^ NFAT1 is a key member of the NFAT family and is involved in the non‐canonical Wnt signaling pathway. It plays a critical role in the regulation of osteogenic differentiation and bone formation.^[^ ^28^ ^]^ Recent studies have indicated that piezoelectric stimulation is closely associated with Piezo1/Ca^2^⁺ signaling, leading to the upregulation of NFAT1 expression in the downstream pathway.^[^ ^29^ ^]^ According to the Western blot results, we confirmed that the expression of Piezo1 and NFAT1 proteins was significantly upregulated in the DAT/KNN + US and DAT/KS + US groups (**Figure** **5**a,b). The Fluo‐4 fluorescence probe was used to detect intracellular calcium ion levels. The piezoelectric composite membranes under ultrasound treatment exhibited higher MFI, indicating an increase in intracellular calcium concentration (Figure 5c,d). Consistently, flow cytometry analysis of Fluo‐4 stained BMSCs in each experimental group revealed that the proportion of labeled cells was 26.1% and 29.5% in the DAT/KNN + US and DAT/KS + US groups, respectively, which were significantly higher than in the other groups (Figure 5e). These results show that piezoelectric stimulation activates Piezo1 protein, allowing calcium influx and increasing the expression of downstream NFAT1 protein. The Wnt/*β*‐catenin signaling pathway is a *β*‐catenin dependent mechanism. Upon activation, *β*‐catenin accumulates in the cytoplasm and translocates to the nucleus. There, it interacts with T cell factor/lymphoid enhancer factor (TCF/LEF) transcription factors to regulate the transcription of target genes.^[^ ^30^ ^]^ The protein expression levels of Wnt/*β*‐catenin signaling related markers were analyzed in each experimental group. Compared to the other groups, the expression of *β*‐catenin protein was significantly upregulated in the DAT/KS and DAT/KS + US groups, especially in the DAT/KS + US group, where the *β*‐catenin expression was the highest. The expression of components of the “destruction complex”, including AXIN2 and GSK‐3*β*, were reduced in the DAT/KS and DAT/KS + US groups, indicating that the DAT/KS membrane protects intracellular *β*‐catenin from degradation (Figure 5f; Figure S19, Supporting Information). Consistently, immunofluorescence staining was performed to observe the expression and localization of *β*‐catenin in the cells, further confirming the high expression of *β*‐catenin in both the cytoplasm and nucleus of the DAT/KS and DAT/KS + US groups (Figure 5g,h).

### 2.6 US‐Driven the DAT/KS Membrane Inhibits Osteoclast Fusion and Function

**Figure 6:**
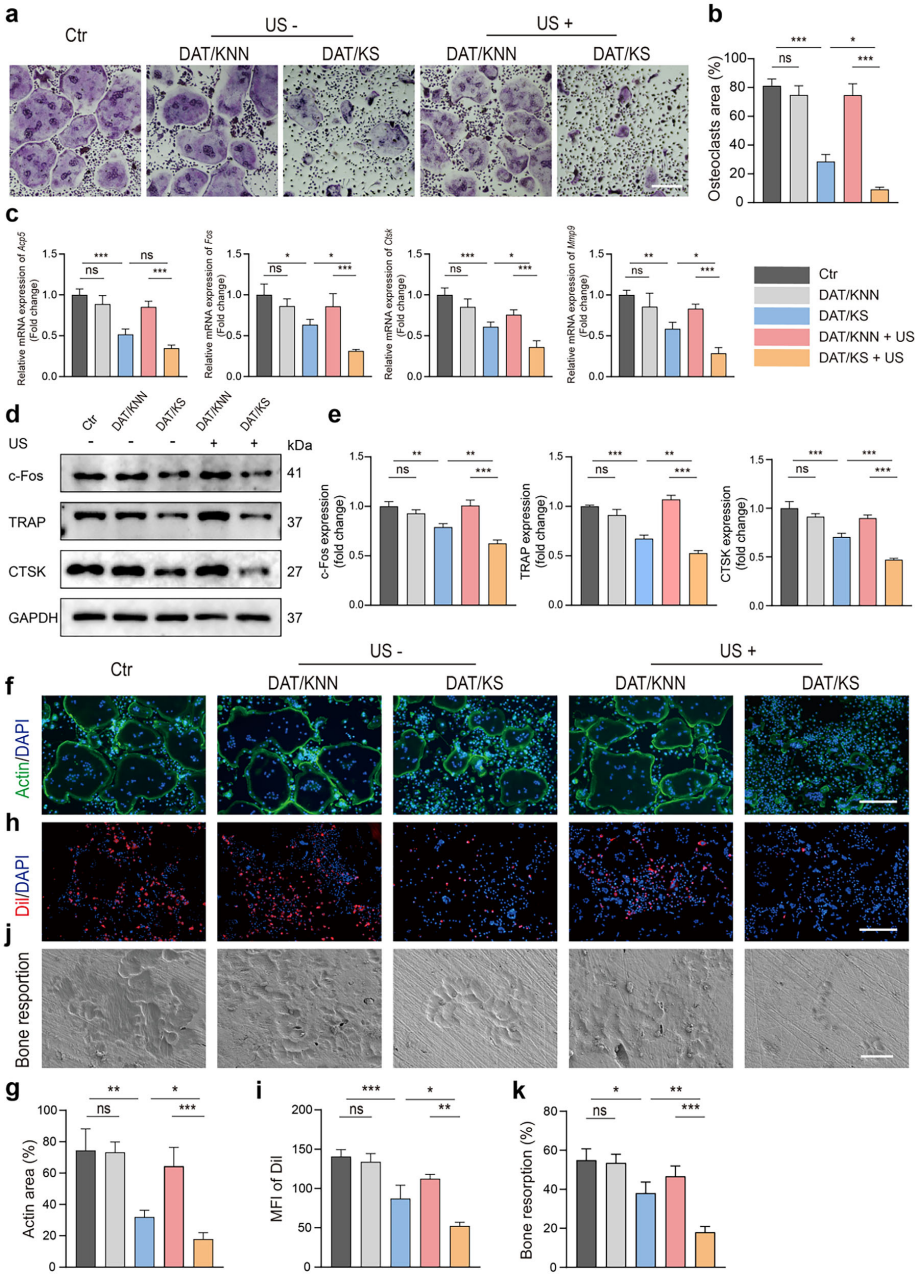
The effects of piezoelectric composite membranes on osteoclast differentiation and bone resorption. a) TRAP staining of osteoclasts in different experimental groups: Ctr, DAT/KNN, DAT/KS, DAT/KNN + US, and DAT/KS + US. b) Semi‐quantitative analysis of osteoclasts area fraction (n = 3, mean ± s.d.). c) Relative mRNA expression levels of osteoclastic differentiation markers (*Acp5*, *Fos*, *Ctsk*, *Mmp9*) in BMMs (n = 3, mean ± s.d.). d) Western blot analysis of osteoclast‐related proteins (c‐Fos, TRAP, CTSK) expression levels in BMMs. e) Semi‐quantitative analysis of c‐Fos, TRAP, and CTSK protein expression levels from Western blot analysis (n = 3, mean ± s.d.). f) Immunofluorescence staining showing Actin (green), and DAPI (blue) in different experimental groups. g) Semi‐quantitative analysis of F‐actin area fraction (n = 3, mean ± s.d.). h) Immunofluorescence staining showing Dil (red), and DAPI (blue) in different experimental groups. i) Semi‐quantitative analysis of Dil MFI (n  =  3, mean ± s.d.). j) SEM images showing bone resorption pits in different experimental groups. k) Semi‐quantitative of bone resorption area fraction (n = 3, mean ± sd). n represents the number of biologically independent samples. Scale bar, 400 µm (a,f,h), 50 µm (j). (*P < 0.05, **P < 0.01, *** *P* < 0.001).

Osteoclasts remove excess bone tissue through bone resorption, playing a key role in bone remodeling, and work in conjunction with osteoblasts to maintain skeletal stability. However, excessive osteoclast activity can lead to osteoporosis and impair fracture healing.^[^ ^4a^ ^]^ To evaluate the effects of the piezoelectric composite membranes on osteoclast differentiation. BMMs co‐cultured with the piezoelectric composite membranes underwent osteoclast differentiation induction with and without ultrasonic treatment. TRAP staining results showed significant osteoclast differentiation in the Ctr, DAT/KNN, and DAT/KS + US groups, while differentiation was notably suppressed in the DAT/KS and DAT/KS + US groups, especially in the DAT/KS + US group. This suggests that the DAT/KS membrane's release of SSD effectively inhibits osteoclast precursor differentiation, particularly under US treatment, where accelerated SSD release resulted in a significant reduction in both the number and area of osteoclast formation (**Figure** **6**a,b). At the molecular level, the mRNA transcription levels of osteoclast differentiation related markers (*Acp5, Fos, Ctsk, Mmp9*) and protein expression levels (TRAP, c‐FOS, CTSK) were significantly reduced in the DAT/KS and DAT/KS + US groups (Figure 6c‐e). According to our previous studies, SSD effectively inhibited the further differentiation of osteoclast precursors by suppressing NF‐κB signaling and P65 nuclear translocation in BMMs.^[^ ^20^ ^]^ Osteoclast differentiation was induced in BMMs from each experimental group. The degradation and phosphorylation levels of IκB*α* were analyzed. In the DAT/KS and DAT/KS + US groups, IκB*α* expression was higher, while its phosphorylation level remained low (Figure S20, Supporting Information). In addition, the phosphorylation level of P65 protein was significantly suppressed in the DAT/KS + US group compared to the other groups (Figure S21, Supporting Information). Immunofluorescence results showed that a higher proportion of BMMs exhibited P65 nuclear translocation in the Ctr, DAT/KNN, and DAT/KNN + US groups, while the proportion of BMMs showing P65 nuclear translocation were reduced in the DAT/KS and DAT/KS + US groups. Notably, the DAT/KS + US group showed the lowest proportion of cells with nuclear translocation (Figures S22 and S23, Supporting Information). Actin and DAPI staining revealed the distribution of the osteoclast cytoskeletal ring and nucleus, with the DAT/KS + US group showing a more effective inhibition of actin ring formation compared to other experimental groups (Figure 6f,g). The DiI red fluorescence probe and bone resorption assays were used to assess the membrane fusion rate and bone resorption activity of osteoclasts. In the DAT/KS and DAT/KS + US groups, the membrane fusion rate and bone resorption pit area of osteoclasts were significantly smaller compared to the CTR, DAT/KNN, and DAT/KNN + US groups. The DAT/KS + US group showed the most effective inhibition (Figure 6h‐k).

### 2.7 Evaluation of Biocompatibility and Angiogenesis In Vivo

**Figure 7:**
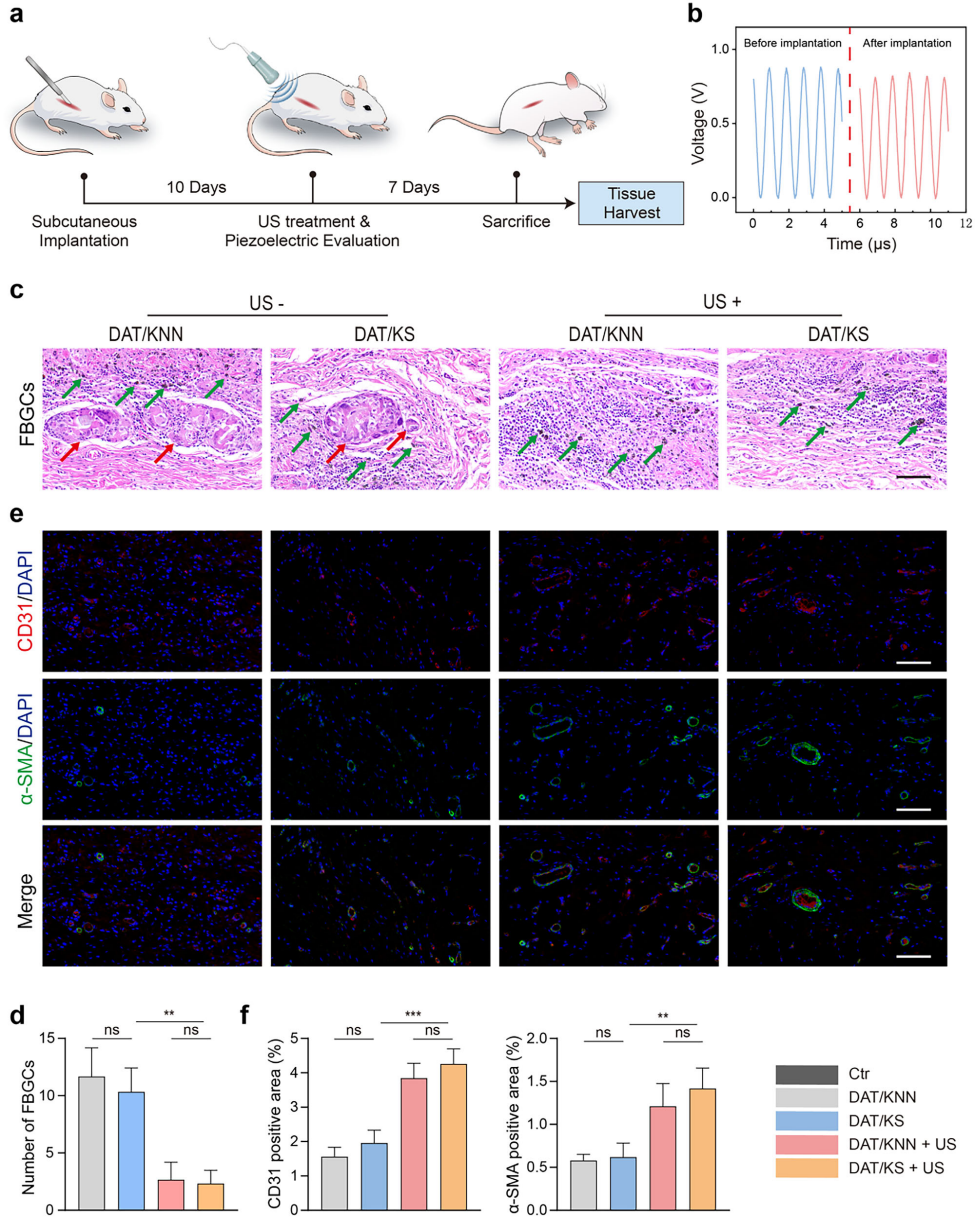
Biocompatibility and angiogenic potential of the piezoelectric composite membranes in vivo. a) Schematic illustration of in vivo subcutaneous implantation, US treatment, and tissue harvest. b) Piezoelectric voltage output of the DAT/KS membranes before and after implantation. c) HE staining of tissue sections from different experimental groups: Ctr, DAT/KNN, DAT/KS, DAT/KNN + US, and DAT/KS + US. Green arrows indicate nanoparticles within the piezoelectric composite membranes, while red arrows highlight FBGCs around the membranes. d) Semi‐quantitative analysis of the number of FBGCs (n = 3, mean ± s.d.). e) Immunofluorescence staining showing CD31 (red), *α*‐SMA (green), and DAPI (blue) in tissue sections from different experimental groups. f) Semi‐quantitative analysis of the positive area of CD31 and *α*‐SMA (n = 3, mean ± s.d.). n represents the number of biologically independent samples. Scale bar, 100 µm (c, e). (*P < 0.05, **P < 0.01, *** *P* < 0.001).

Biomaterials with excellent biocompatibility reduce inflammatory infiltration, promote the recruitment and activation of repair cells, and ensure adequate blood supply to the damaged tissue, thereby accelerating healing and functional recovery. **Figure** **7**a illustrates the schematic of the subcutaneous implantation experiment, where the piezoelectric composite membrane was implanted into the dorsal region of rats. US treatment began on day 10 post‐surgery and continued for 7 days. At the end of the experimental period, skin tissue surrounding the implanted membrane was collected for histological staining to assess in vivo biocompatibility and angiogenic potential. Compared to the in vitro piezoelectric output, the implanted piezoelectric composite membrane maintained stable voltage output under 0.3 W cm^−^^2^ US intensity (Figure 7b). HE staining of tissue sections revealed black nanoparticles within the piezoelectric composite membrane, with significant infiltration of inflammatory cells. Furthermore, foreign body giant cells (FBGCs) formation was observed in the DAT/KNN and DAT/KS groups, whereas the number of foreign body giant cells were significantly reduced in the DAT/KNN + US and DAT/KS + US groups, indicating that piezoelectric stimulation further alleviated the local foreign body reaction (Figure 7c,d). Additionally, we evaluated the influence of the piezoelectric composite membrane on red blood cells through hemolysis assays. After incubating the piezoelectric composite membrane with rat red blood cell suspension at 37 °C for 4 h, hemolysis rates in the DAT/KNN and DAT/KS groups were both below 5%, indicating good biocompatibility of the membrane (Figure S24, Supporting Information).

**Figure 8:**
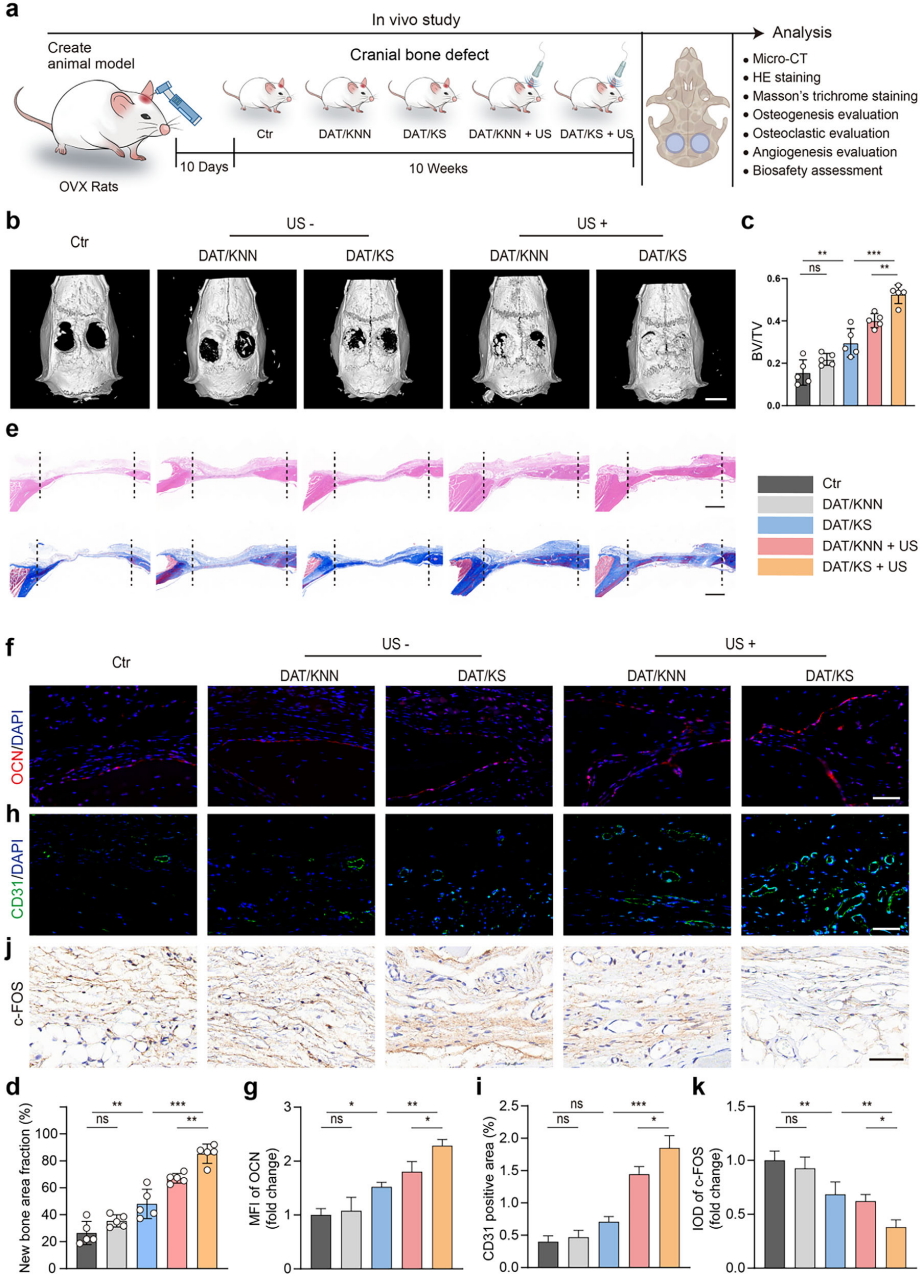
Potential of the piezoelectric composite membranes for the repair of osteoporotic bone defects. a) Schematic illustration of osteoporotic bone defect model, piezoelectric composite membrane implantation, US treatment, and cranial tissue collection for evaluation. b) Micro‐CT images of the bone defect region in different experimental groups: Ctr, DAT/KNN, DAT/KS, DAT/KNN + US, and DAT/KS + US. c) Quantitative analysis of BV/TV (n = 5, mean ± s.d.). d) Semi‐quantitative analysis of new bone area fraction(%) (n = 5, mean ± s.d.). e) HE and Masson's trichrome staining of tissue sections from different experimental groups. f) Immunofluorescence staining showing OCN (red) and DAPI (blue) in tissue sections from different experimental groups. g) Semi‐quantitative analysis of OCN MFI (n = 3, mean ± s.d.). h) Immunofluorescence staining showing CD31 (green) and DAPI (blue) in different experimental groups. i) Semi‐quantitative analysis of the positive area of CD31 (n = 3, mean ± s.d.). j) Immunohistochemistry showing c‐FOS expression in tissue sections from different experimental groups. k) Semi‐quantitative analysis of c‐FOS integratedoption density (IOD) (n = 3, mean ± s.d.). n represents the number of biologically independent samples. Scale bar, 5 mm (b), 1 mm (e), 50 µm (f,h,j). (*P < 0.05, **P < 0.01, *** *P* < 0.001).

Angiogenesis plays a crucial role in bone tissue repair by facilitating nutrient supply and waste removal.^[^ ^31^ ^]^ CD31 and *α*‐SMA serve as markers for endothelial and smooth muscle cells, respectively. Their coordinated action is essential for angiogenesis and vascular function.^[^ ^32^ ^]^ Therefore, we also evaluated the impact of the piezoelectric composite membrane on vascularization. Immunofluorescence staining results showed a significant upregulation of CD31 and *α*‐SMA expression in the DAT/KNN + US and DAT/KS + US groups, with a large proportion of positive staining areas (**Figure** **8**e,f). This suggests that piezoelectric stimulation promotes vascular regeneration around the membrane, accelerates nutrient metabolism, and ultimately facilitates tissue healing.

### 2.8 Regeneration of Osteoporotic Bone Defects In Vivo

Figure 8a presents an in vivo study investigating the effects of US‐driven piezoelectric composite membranes on bone regeneration in osteoporotic conditions. First, bone mass was evaluated in ovariectomized (OVX) rats. The microCT 3D imaging results showed that, compared to the normal rat, the distal femoral cortical bone in OVX rat was thinner, with sparser trabeculae and a significant reduction in bone mass, confirming the successful establishment of the osteoporotic model (Figure S25, Supporting Information). Subsequently, two critical circular defects (5 mm in diameter) were created symmetrically along the cranial suture of OVX rats, and the piezoelectric composite membranes were implanted into these defect sites (Figure S26, Supporting Information). 10 days after surgery, these rats in the DAT/KNN + US and DAT/KS + US groups underwent US treatment (Figure S27, Supporting Information). The rats were maintained on a normal diet for ≈10 weeks before being euthanized for tissue sampling. As shown in Figure 8b, the critical cranial defects in the DAT/KS + US group were nearly fully repaired with newly formed bone. In the DAT/KNN + US group, cranial defects were partially repaired, while substantial defects remained in the Ctr, DAT/KNN, and DAT/KS groups. The bone volume/total volume (BV/TV) and the proportion of newly formed bone were significantly higher in the DAT/KS + US group compared to the other groups (Figure 8c,d).

The osteogenic, angiogenic, and osteoclastic inhibition in vivo were assessed through HE, Masson's Trichrome, and immunohistochemical analysis of OCN, CD31, and c‐FOS. HE staining showed significant new bone formation in the DAT/KS + US group, along with extensive matrix production in the defect area. Masson's Trichrome staining demonstrated a rich and dense collagen matrix in the DAT/KS + US group, suggesting active osteogenesis, whereas only limited or sparse collagen formation was observed in the other groups (Figure 8e). Consistently, immunofluorescence staining of OCN demonstrated significantly higher fluorescence intensity in the DAT/KS + US group compared to the other groups, indicating enhanced osteogenic differentiation of bone tissue (Figure 8f,g). Bone tissue regeneration is accompanied by neovascularization. Immunofluorescence staining of bone tissue sections was performed using the endothelial cell marker CD31, with the DAT/KS + US group showing the strongest fluorescence intensity (Figure 8h). Semi‐quantitation analysis results showed that the CD31 area fraction in the DAT/KS + US group was significantly higher than in the other groups (Figure 8i). In the pathological process of osteoporosis, excessive osteoclast activity leads to greater bone resorption than bone formation, complicating bone repair.^[^ ^33^ ^]^ In pathological bone resorption, the overexpression and activation of c‐FOS is a key mechanism contributing to bone loss.^[^ ^34^ ^]^ Immunohistochemical analysis of bone tissue sections from the bone defect area revealed that c‐FOS expression was significantly lower in the DAT/KNN + US group compared to the other groups. This suggests that the DAT/KNN + US membrane, under ultrasonic treatment, accelerates bone formation and inhibits bone resorption through piezoelectric stimulation and SSD release, ultimately restoring bone homeostasis and facilitating bone defect repair under pathological conditions (Figure 8j,k).

In addition, we harvested and analyzed the hearts, kidneys, livers, lungs, and spleens of experimental rats from each group. The results shows no significant differences between groups, further confirming that the piezoelectric composite membranes and US treatment were biologically safe and non‐toxic to the animals (Figure S28, Supporting Information).

## 3 Conclusion

This study presents an US‐responsive piezoelectric composite membrane with excellent biocompatibility, mechanical strength, and degradability. The DAT/KS membrane successfully enables bidirectional regulation of osteogenic and osteoclastic differentiation, restoring bone homeostasis imbalance in osteoporotic background. By controlling electrical signal output and SSD release, the DAT/KS membrane enhances cellular energy status, promoting the recruitment of BMSCs to the healing site during the early stages, thereby laying the groundwork for subsequent bone repair. Under US stimulation, the DAT/KS membrane accelerates osteoblast differentiation and mineralization while inhibiting osteoclast fusion and bone resorption, thereby demonstrating superior regenerative effects in osteoporotic bone repair through this bidirectional regulation strategy. Mechanistically, activation of both canonical and non‐canonical Wnt signaling in BMSCs drives their osteogenic differentiation fate, while the efficient release of SSD inhibits the NF‐κB pathway in BMMs, limiting osteoclast differentiation and function to alleviate excessive osteoclast activity in osteoporotic background. Overall, we have implemented the “two‐way regulation” bone homeostasis strategy using external field‐responsive piezoelectric composite materials, providing a key foundation for further research on medical interventions for bone injuries under osteoporotic conditions.

## 4 Experimental Section

### Materials

Niobium hydroxide oxide (Nb(OH)₅), oxalic acid, sodium carbonate (Na₂CO₃), potassium carbonate (K₂CO₃), isopropyl alcohol, triacylglycerol lipase, 2‐amino‐2‐hydroxymethylpropane‐1,3‐diol (Tris buffer) and ethylenediaminetetraacetic acid buffer (EDTA buffer) were purchased from Aladdin (Shanghai, China). Saikosaponin D (SSD, 98.8%) was supplied from MedChemExpress (MCE, Shanghai, China). Cell Counting Kit‐8, live/dead assay kit, Cell Cycle and Apoptosis Analysis Kit, Alkaline Phosphatase Assay Kit, Alizarin Red S staining solution, Enhanced mitochondrial membrane potential assay kit with JC‐1, 1,1′‐dioctadecyl‐3,3,3′,3′‐tetramethylindocarbocyanine perchlorate (Dil staining), Actin‐Tracker Red‐555, Actin‐Tracker Green‐488 and 6′‐diamidino‐2‐phenylindole (DAPI) were obtained from Beyotime Biotechnology (Shanghai, China). *α*‐Minimum essential medium (*α*‐MEM) and fetal bovine serum (FBS) were obtained from Gibco (USA). All chemicals were employed in their original form without undergoing any additional purification steps.

### Preparation of KNN Nanoparticles and KNN‐SSD Nanoparticles

In brief, a certain amount of oxalic acid was dissolved in distilled water, and then Nb(OH)₅ powder (14.4 g) was added, achieving a molar ratio of 1:8 between niobium hydroxide and oxalic acid. The solution was stirred at 80 °C until complete dissolution, resulting in a niobium‐containing oxalic acid solution. Na₂CO₃ (0.53 g) and K₂CO₃ (0.69 g) were successively dissolved into this solution, and the pH was adjusted to 4 using ammonia solution. Ethylene glycol (100 mL) was then added, and the mixture was stirred at 90 °C until the solution turned light yellow. Once the solution became viscous, it was placed in a drying oven at 115 °C to dry, yielding a white gel. The dried gel was subsequently treated at 400 °C for 2 h and then calcined at 600 °C for 2 h to obtain a white solid powder. Finally, after grinding, washing, and centrifuging, the resulting sodium potassium niobate (KNN) nanoparticles were dried at 60 °C for 12 h. The KNN nanoparticles were then subjected to hydroxylation and surface modification with SSD. The KNN nanoparticles (1.5 g) were added to a hydrogen peroxide solution (100 mL) and placed in a 250 mL round‐bottom flask. The mixture was subjected to ultrasonic treatment for 20 min to ensure the full dispersion of the KNN nanoparticles. Subsequently, the solution was refluxed in a silicone oil bath preheated to 105 °C for 4 h to obtain hydroxylated KNN nanoparticles. The hydroxylated KNN nanoparticles were then mixed with SSD (solid‐state deposition) in ultrapure water and stirred for 6 h. Finally, the mixture was subjected to centrifugation and washing, followed by vacuum drying to obtain the modified KNN‐SSD nanoparticles.

### Preparation of DAT

Subcutaneous fat from pigs was processed under sterile conditions. Following previously reported decellularization protocols,^[^ ^29b^ ^]^ the tissue underwent three freeze‐thaw cycles (−80–37 °C) in a cold buffer with 10 mM Tris and 5 mM EDTA. The tissue was homogenized with a grinder and washed three times with PBS. Next, it was incubated overnight at 37 °C at 1000 rpm in an enzymatic solution with 0.25% trypsin and 0.02% EDTA. The tissue was then extracted for 12 h with 99.9% isopropanol to remove lipids. After three PBS washes, it was further incubated for 4 h in the same enzymatic solution. Subsequently, the tissue was incubated for 6 h at 37 °C in a solution with 5000 U DNase, 10 mg RNase, and 2000 U lipase. After three PBS washes, it was extracted for 8 h with 99.9% isopropanol. Following three additional PBS washes, 75% ethanol was used for final homogenization. The resulting DAT was filtered under vacuum to form a membrane, air dried at room temperature, and stored under vacuum for later use. The DAT membrane remains stable at 4 °C for up to 6 months.

### Immunohistochemical Analysis and DNA Quantification of DAT

The native and decellularized tissues were fixed in 4% paraformaldehyde and embedded in paraffin for sectioning. The sections were deparaffinized and dehydrated in graded ethanol. Hematoxylin‐eosin (HE) and Masson's trichrome staining were used to assess residual cells and collagen deposition. Oil Red O staining was used to assess lipid content. Sections were stained with DAPI for 20 min at room temperature to visualize nucleic acids. The sections were examined using a fluorescence microscope (Leica, Germany). To assess decellularization efficiency, genomic DNA was extracted from native and decellularized tissues using a DNA Extraction Kit (Tiangen Biotech, Beijing, China) following the manufacturer's protocol. The DNA concentration was measured using a NanoDrop 2000 (Thermo Fisher Scientific, USA).

### Fabrication of the DAT/KS Membrane

The DAT membrane was immersed in ultrapure water and homogenized using a tissue grinder to form a tissue suspension. Next, The KNN‐SSD nanoparticles with different mass ratios (10%, 20%, 30%) were then added to the suspension and thoroughly mixed. After vacuum filtration, a piezoelectric composite membrane was constructed and dried in an oven at 37 °C. The final piezoelectric composite membrane was stored under vacuum for subsequent experiments.

### Characterization of the Membranes and Piezoelectricity

The morphologies and chemical composition of nanoparticles and the DAT/KS membrane were characterized by scanning electron microscopy (SEM, ZEISS GeminiSEM 300, Germany), transmission electron microscopy (TEM, Tecnai G2 F20, Netherlands), and Fourier transform infrared spectroscopy (FTIR, Thermo Scientific Nicolet iS20, USA). The phase compositions of KNN and KNN‐SSD nanoparticles were identified using X‐ray Diffraction (XRD, smartlab, Japan). The UV–vis measurements were conducted at room temperature using a UV–vis spectrophotometer (Thermo Scientific, USA). The particle size distribution and the zeta potentials of nanoparticles and DAT/KS membrane were achieved from the dynamic light scattering (Malvern, UK). The valence state of the DAT/KS membrane was determined by X‐ray photoelectron spectroscopy (XPS, Thermo ESCALAB 250XI, USA). AFM measurement was implemented on an SPM9700 scanning probe microscope (Bruker Dimension ICON, USA) to reveal the 3D structure of the composite films.

The piezoelectric coefficient *d*_33_ values of samples were measured by a quasi‐static *d*_33_ meter (ZJ‐6). The piezoelectric output characteristics of the samples were measured using an oscilloscope (MDO 32, Tektronix) under ultrasonic stimulation at 1 MHz, generated by a therapeutic US device (WED‐100, WELLD), with all tests conducted at ambient temperature. The packaging and voltage output measurement of the piezoelectric DAT/KS membrane were performed using previously reported methods.^[^ ^35^ ^]^ Specifically, the DAT/KS membrane (25 mm × 25 mm, thickness, 0.13 µm) was sandwiched between two copper foil electrodes. Then, wires were connected to the copper foil electrodes using conductive silver adhesive, and finally, the entire device was encapsulated in a PI film. The piezoelectric performance of DAT/KS membranes with different KNN‐SSD nanoparticles mass ratios (10%, 20%, 30%) was studied under a power density of 0.3 W cm^−^^2^. Additionally, different US intensities (0.1 W cm^−^^2^, 0.3 W cm^−^^2^, 0.5 W cm^−^^2^) were applied to the DAT/20%KS membrane, and the electrical signal output was measured. The piezoelectric properties were visualized using Origin software (OriginLab, USA).

### In Vitro Biodegradation

The degradability of the membranes were tested by incubating it in PBS solution (pH 7.4) containing 5 CDU mL^−1^ collagenase. To ensure continuous enzyme activity, the collagenase solution was refreshed daily. Samples were taken from the solution every two days, rinsed with deionized water, freeze‐dried, and weighed. Each membrane was tested in triplicate. The extent of degradability was determined by calculating the percentage of the remaining dry weight of the membrane after collagenase treatment.

(1) $$\mathrm{Remaining}\;\mathrm{weight}\;\left(\%\right)=\left(\frac{W_{d}}{W_{0}}\right)\times 100$$

Wo represents the initial weight of the scaffolds, while Wd denotes the remaining weight at different time points (n  =  3, mean ± s.d.).

### Cell Culture

All animal experiments were carried out according to protocols approved by the Animal Care and Use Committee of Shanghai Tenth People's Hospital, School of Medicine, Tongji University, Shanghai, China (ethical approval number: SHDSYY‐2024‐2530‐G3). BMSCs were isolated from the femur and tibia of 4‐week‐old male Sprague Dawley (SD) rats from Shanghai Laboratory Animal Center (SLAC, China). BMSCs were cultured in *α*‐MEM with 10% fetal bovine serum (FBS) and 1% penicillin/streptomycin (PS) (Gibco Invitrogen, USA) at 37 °C in 5% CO₂. For osteogenic differentiation, the medium was replaced with complete *α*‐MEM containing 50 µg mL^−1^ L‐ascorbic acid, 10 mM *β*‐glycerophosphate, and 10 nM dexamethasone (Sigma Aldrich, USA) every two days. Fresh BMMs from 8‐week‐old C57BL/6J mice were isolated as previously reported.^[^ ^36^ ^]^ The bone marrow from tibia and femur was flushed and cultured in complete *α*‐MEM. To obtain pure BMMs, non‐adherent cells were collected and cultured in complete *α*‐MEM with 40 ng mL^−1^ M‐CSF (Amizona, USA). After three days of culture, adherent cells were used for experiments. BMMs were then stimulated with 40 ng mL^−1^ M‐CSF and 40 ng mL^−1^ RANKL (Amizona, USA) for five days to generate mature osteoclasts.

### Cell Apoptosis Evaluation

Cell apoptosis was detected using flow cytometry. BMSCs were seeded in a 6‐well plate at a density of 2 × 10^5^ cells per well. After 6 h of incubation, the DAT/KS membranes was applied to culture with the cells under different US intensities (0.1 W cm^−^^2^, 0.3 W cm^−^^2^, 0.5 W cm^−^^2^). After 2 days, an Annexin V‐FITC Apoptosis Detection Kit was used to assess apoptosis according to the manufacturer's instructions. The apoptosis rate was quantified by flow cytometry. Annexin V‐negative and PI‐positive cells were considered as mechanically damaged cells, while Annexin V‐positive and PI‐positive cells were classified as late apoptotic cells.

### Mitochondrial Membrane Potential Detection

JC‐1 dye was used to determine mitochondrial membrane potential in BMSCs. Briefly, the JC‐1 staining working solution was prepared according to the manufacturer's instructions and set aside. For the DAT/KNN + US and DAT/KS + US groups, US stimulation was applied. The culture medium was then aspirated from the 24‐well plate, and the cells were gently washed once with PBS. Next, 500 µL of complete *α*‐MEM was added, followed by 500 µL of the JC‐1 staining solution, which was thoroughly mixed. The cells were incubated at 37 °C for 20 min. After incubation, the supernatant was removed, and the cells were washed twice with staining buffer. Finally, 1 ml of complete *α*‐MEM was added to each well. The mitochondrial membrane potential of cells in each group was observed under a fluorescence microscope (Leica DMI6000B, Germany).

### Cell Biocompatibility and Proliferation

The biocompatibility of the DAT/KNN and DAT/KS membranes were assessed via a live/dead assay. BMSCs were seeded in 24‐well plates and incubated for 6 h for full attachment. Sterilized piezoelectric composite membranes from each group were placed over the wells for complete coverage. In the DAT/KNN + US and DAT/KS + US groups, US was applied for 20 min daily. Before US treatment, US gel was applied to the well bottoms to enhance sound wave transmission. US was delivered in 5 minute cycles with 1 minute pauses to prevent localized overheating that could impair cell viability. After 48 h, cell viability was assessed using a live/dead assay kit under the fluorescence microscope, following the manufacturer's instructions. For cell proliferation analysis, BMSCs were seeded in 96‐well plates for 6 h before being covered with the DAT/KNN or DAT/KS membranes. In the US treatment group, ultrasonic treatment was appldaily. At days 1, 3, and 5, the membranes were removed, and cell proliferation was assessed using the CCK‐8 kit. After removing the medium and washing three times with PBS, 100 µL of serum‐free medium and 10 µL of CCK‐8 solution were added per well. After 2 h at 37 °C, absorbance at 450 nm was measured using a microplate reader (Thermo Fisher Scientific, USA). Based on these results, the DAT/20%KS membrane was selected for further experiments. Unless stated otherwise, DAT/KS refers to the DAT/20%KS membrane.

### In Vitro Assay for Osteoblast Differentiation and Function

To assess the effects of piezoelectric stimulation on osteoblast differentiation, BMSCs were co‐cultured with the piezoelectric membranes in osteogenic medium with or without US treatment for 14 days. ALP activity was evaluated using an Alkaline Phosphatase Assay Kit. Cells in each well were washed with PBS and fixed in 4% paraformaldehyde for 15 min. After three PBS washes, BCIP/NBT solution was added to each well and incubated at room temperature for 30 min. The cells were rinsed three times with PBS and observed under a microscope. ALP activity was quantified by analyzing the stained area using ImageJ software (National Institutes of Health, USA).

To evaluate the formation of mineralized matrix nodules during osteogenic differentiation, BMSCs were cultured in osteogenic medium with or without US for 21 days. Mineralized nodule formation was assessed using the Alizarin Red S Staining Kit, following the manufacturer's instructions. Cells in each well were briefly washed with PBS and fixed with 4% paraformaldehyde for 15 min. After three PBS washes, Alizarin Red S solution was added to each well and incubated at room temperature for 30 min. The cells were rinsed twice with PBS and observed under a microscope. For quantification of mineralization, mineralized bone nodules were eluted with 10% cetylpyridinium chloride and measured by spectrophotometry at 562 nm using a microplate reader.

### Measurement of Intracellular Calcium Levels

Intracellular calcium levels were assessed using Fluo‐4 direct calcium assay kits (Beyotime, China). After 20 min of US treatment, the culture medium was discarded, the wells were washed three times with PBS, then 100 µL of Fluo‐4 Staining Solution was added, and the cells were incubated at 37 °C for 30 min in the dark. The stained cells were then observed under a fluorescence microscope, and the mean fluorescence intensity was quantified using ImageJ software. Similarly, cells in another 6‐well plate co‐cultured with DAT/KNN and DAT/KS membranes were exposed to US treatment. After trypsin digestion without EDTA, cells were collected, washed with PBS, and stained with Fluo‐4 Staining Solution. Finally, the cells in each well were resuspended in PBS and analyzed by flow cytometry.

### In Vitro Assay for Osteoclast Fusion and Function

To assess the effects of the DAT/KNN and DAT/KS membranes on osteoclast differentiation under US stimulation, BMMs were seeded at 1 × 10⁵ cells per well in 24‐well plates and cultured in *α*‐MEM with 40 ng mL^−1^ M‐CSF. After 6 h, sterilized DAT/KNN and DAT/KS membranes were placed over the wells for full coverage. RANKL (40 ng mL^−1^) was added to each well on days 1, 3, and 5 to promote osteoclastogenesis. The DAT/KNN + US and DAT/KS + US groups received 20 min of daily US stimulation. When mature osteoclasts were observed in the control group, TRAP (Tartrate‐Resistant Acid Phosphatase, Amizona, USA) staining and DiI staining were used to analyze osteoclast distribution and pre‐osteoclast membrane fusion rate. The areas of multinucleated osteoclasts (>3 nuclei) and mononucleated TRAP‐positive cells were quantified. DiI red fluorescence images were captured with a fluorescence microscope, and mean fluorescence intensity was quantified using ImageJ software.

Freeze‐dried beef bone slices were sterilized by soaking in 75% ethanol for 2 h, washed three times with 0.9% NaCl solution, and used to assess bone resorption. BMMs were seeded on bone slices and induced to differentiate as previously described.^[^ ^37^ ^]^ The DAT/KNN + US and DAT/KS + US groups received daily US treatment. On day 12 of induction, the bone slices were cleared of medium. Cells were dissociated with a cell dissociation solution (Sigma‐Aldrich, USA) and scraped from bone slices with a brush. Bone slices were sputter‐coated with Au‐Pd and imaged under an SEM.

### qPCR Assay

Total RNA was isolated from cells using Total RNA Extraction Reagent (TRIzol, Invitrogen, USA). The RNA was reverse‐transcribed into cDNA with reverse transcription reagents (Vazyme, China). Here, quantitative PCR (qPCR, Vazyme, China) and qPCR with reverse transcription systems were used according to the manufacturer's instructions. Osteogenic‐related mRNA markers (Rat‐derived) of *Alpl, Bglap, Col1a1* and *Runx2*, and osteoclast‐related mRNA markers (mouse‐derived) of *Acp5, Ctsk, Fos* and *Mmp9* were calculated using the 2^−ΔΔCt^ method. Lists of the primer sequences used for qPCR in this study are provided in TableS1 and TableS2.

### Western Blot Analysis

Protein extraction was performed using RIPA lysis buffer. SDS–PAGE gels were used to separate the extracted proteins. After electrophoresis, polyvinylidene difluoride (PVDF) membranes were employed for protein transfer. Proteins were blocked with 5% BSA in TBST. After incubation with primary and secondary antibodies, chemiluminescent signals were detected using enhanced chemiluminescence reagents (Vazyme, China).

### Evaluation of the Biocompatibility and Angiogenesis of the Membranes

A subcutaneous membrane implantation model was developed in rats to assess biocompatibility and angiogenesis induced by piezoelectric effects. In brief, twelve 12‐week‐old male Sprague Dawley (SD) rats were anesthetized with pentobarbital. The middle area on the back was shaved and disinfected with iodine, and a subcutaneous pocket was made to create 1.5 cm long vertical incisions. DAT/KNN or DAT/KS membranes were inserted, and the wounds were sutured. From the 10th day post‐surgery, daily US treatment was applied on the implantation area under isoflurane anesthesia for 30 min (1.0 MHz, 0.3 W cm^−^^2^, 10 ms pulse duration). After 7 days, the rats were euthanized, and the next experimental step was performed. The piezoelectric effect of the DAT/KS membrane under treatment stimulation, both in vitro and in subcutaneous tissues, was measured with the Keithley 6514 electrometer.

Samples were dehydrated, embedded in paraffin, and sectioned for histological staining. Serial 5 µm subcutaneous tissue sections underwent H&E and immunofluorescent staining. H&E staining followed the manufacturer's protocol. Inflammatory cell infiltration and foreign body giant cells (FBGCs) were examined under a microscope to assess the biocompatibility of DAT/KNN and DAT/KS membranes under piezoelectric effects. Paraffin sections were deparaffinized and underwent antigen retrieval with a one‐step dewaxing/antigen retrieval buffer. Sections were incubated with angiogenesis‐related antibodies (CD31, *α*‐SMA), followed by secondary antibodies and DAPI. Sections were scanned and recorded with an automatic digital slide scanner. The fluorescence area ratio was quantified with Image J software.

### Evaluation of the Repair Capacity for Osteoporotic Bone Defects

To establish an osteoporosis rat model, 12‐week‐old female Sprague‐Dawley rats underwent an ovariectomy operation. After 8 weeks of scientific feeding, rats in the OVX group and the normal group were separately sacrificed for the right femur. To assess the osteogenic efficacy of piezoelectric composite membranes with or without US, a 5 mm critical‐sized cranial defect was created in OVX rats. Thirty rats were randomly divided into five groups: i) Ctr (unfilled), ii) DAT/KNN, iii) DAT/KS, iv) DAT/KNN + US, and v) DAT/KS + US. Rats were anesthetized with sodium pentobarbital via intraperitoneal injection. The surgical site was shaved and disinfected with iodine. A full‐thickness incision was made along the sagittal suture, and two 5 mm bone defects were drilled into both parietal bones. The DAT/KNN and DAT/KS membranes were placed over the bone defects. The incision was then closed in layers. Postoperatively, rats were allowed free movement, and penicillin was administered for 1 week to prevent infection. In the ultrasonic treatment groups, US treatment (1.0 MHz, 0.3 W cm^−^^2^, 10 ms pulse duration) began on the 10th postoperative day under isoflurane anesthesia for 30 min, every other day. At the end of the experiment, rats were euthanized with an intravenous overdose of 3% pentobarbital sodium. Skulls were harvested and preserved in 4% paraformaldehyde for further analysis.

### Micro–Computed Tomography

New bone formation was assessed by scanning the collected skulls with a micro‐CT system (NMC‐200, NEMO, China). The region of interest (ROI) was defined as a 5 mm diameter, 2 mm depth cylinder for bone regeneration analysis, followed by 3D sample reconstruction. Parameters such as bone volume (BV; mm^3^) and total volume (TV; mm^3^) were analyzed.

### Histology and Immunohistochemistry

After micro‐CT scanning, samples were decalcified, dehydrated through graded ethanol, and embedded in paraffin. Then, sections of ≈5 µm were prepared for further staining. HE and Masson's trichrome staining were performed to evaluate bone formation and residual materials. Immunofluorescence staining was used to assess the expression of osteogenic differentiation protein (OCN) and local angiogenesis marker (CD31). c‐FOS expression was assessed using immunohistochemistry to evaluate local osteoclast activity. Major organs including hearts, kidneys, livers, lungs, and spleens were collected and stained with HE to assess the in vivo toxicity of implanted piezoelectric composite membranes. All samples were imaged with both an optical and fluorescence microscope. Related Proteins expressions were quantified using ImageJ software.

### Statistical Analysis

All experiments were independently performed at least three times and data were expressed as the mean ± standard deviation. Statistical comparisons of two independent groups were performed using unpaired two‐tailed t‐test. Multiple comparisons were performed using one‐way analysis of variance (ANOVA) with post‐hoc Tukey test. Statistical analyses were performed using the GraphPad Prism 9 software (GraphPad Software, Inc., USA). Differences were considered significant at *P < 0.05 and **P < 0.01, highly significant at ***P < 0.001, and not significant at *P* > 0.05 (ns).

## Conflict of Interest

The authors declare no conflict of interest.

## Supporting information



Supporting Information

## Data Availability

The data that support the findings of this study are available from the corresponding author upon reasonable request.
