# Supplementary material for: Ultrasound‐Responsive Piezoelectric Membrane Promotes Osteoporotic Bone Regeneration via the “Two‐Way Regulation” Bone Homeostasis Strategy
Source: Adv Sci (Weinh). 2025 Apr 28;12(27):2504293. doi: 10.1002/advs.202504293 (PMC12279238; doi:10.1002/advs.202504293)
Supplement: Supplementary file 1 — Supporting Information [file ADVS-12-2504293-s001.docx]

**Supporting Information**

**Ultrasound-Responsive Piezoelectric Membrane Promotes Osteoporotic Bone Regeneration via the "Two-Way Regulation" Bone Homeostasis Strategy**

Xinhui Wu^1#^, Tianlong Wang^1#^, Jinhui Zhao^1#^, Lei Zhang^1^, Zhiqing Liu^1^, Yixing Chen^1^, Yiping Luo^1^, Yaqi Liu^1^, Yan Chen^1^, Hui Jiang^1^, Dilixiati Duolikun^1^, Junjian Liu^1^*, Wentao Cao^4^*, and Longpo Zheng^1,2,3^*

^1^Department of Orthopedics, Shanghai Tenth People’s Hospital, School of Medicine, Tongji University, Shanghai 200072, China.

^2^Shanghai Trauma Emergency Center, Shanghai 200072, China.

^3^Orthopedic Intelligent Minimally Invasive Diagnosis & Treatment Center, Shanghai Tenth People's Hospital, Tongji University School of Medicine, Shanghai 200072, China.

^4^Department of Prosthodontics, Shanghai Stomatological Hospital & School of Stomatology, Fudan University, Shanghai 201102, P. R. China.

Emails: jjliu@tongji.edu.cn; wt_cao@fudan.edu.cn; dr.zheng@tongji.edu.cn

Contents

1. Supplementary Figure (Figure S1 – Figure S28)

2. Supplementary Table (Table S1 – Table S2)

**
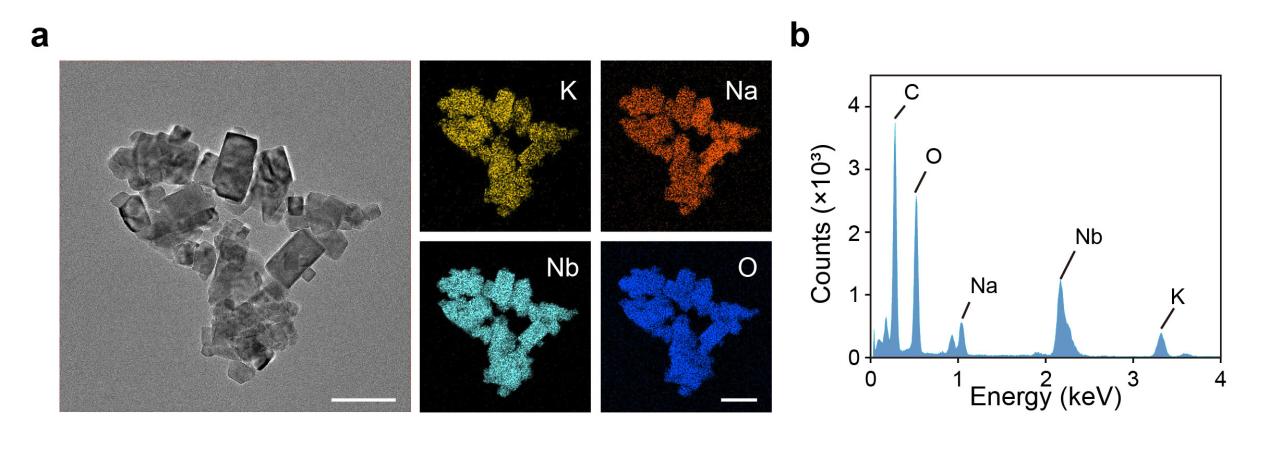
**

**Figure S1. Characterization of KNN nanoparticles.** a) TEM image and EDX elemental mapping images of KNN nanoparticles. b) Corresponding EDX spectral analysis. Scale bar, 100 nm.


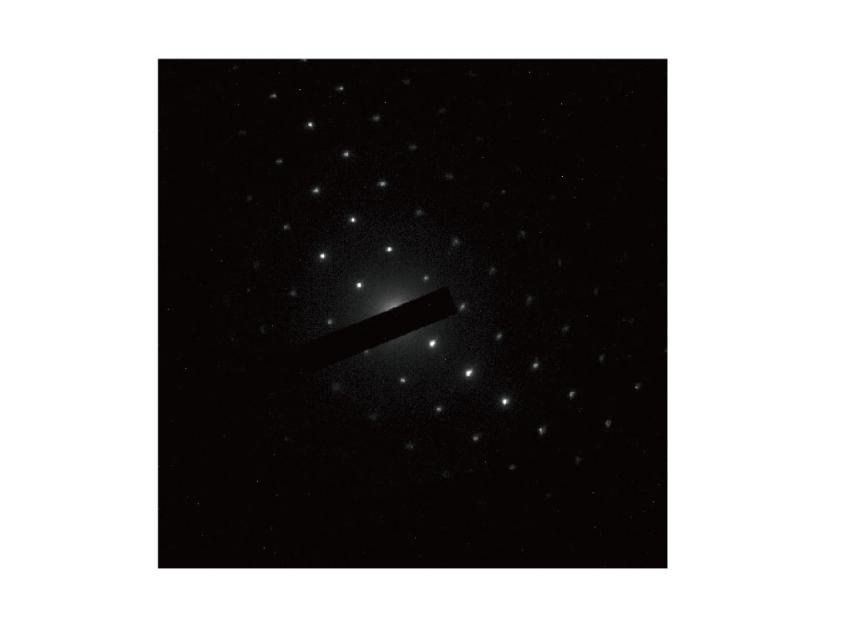


**Figure S2. The corresponding diffraction signal of KNN nanoparticles.**

**
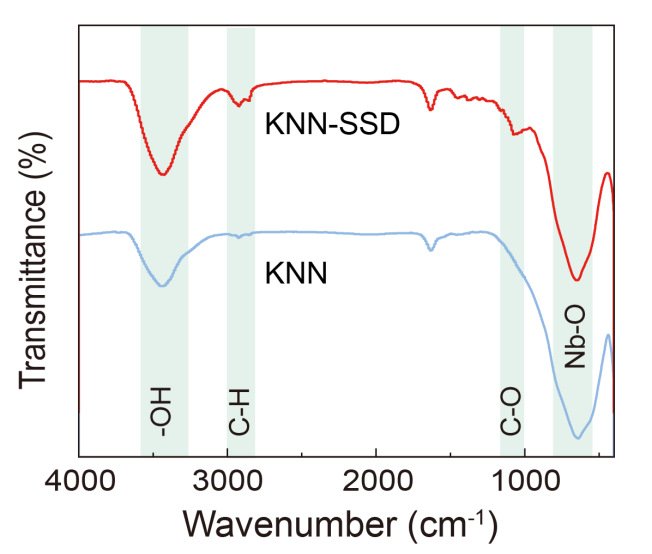
**

**Figure S3. FTIR spectra of KNN and KNN-SSD nanoparticles.**


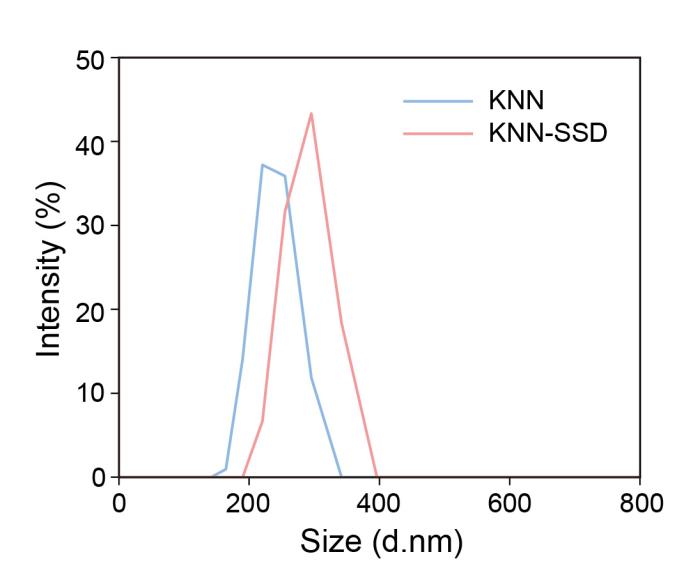


**Figure S4. The size distribution of KNN and KNN-SSD nanoparticles.**

**
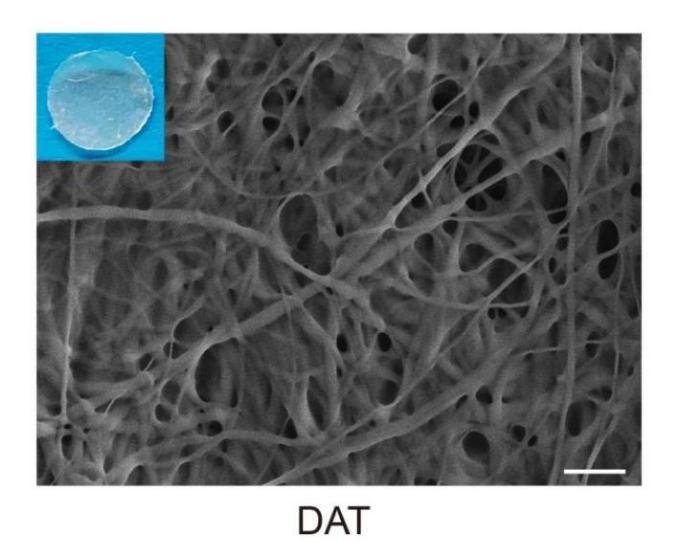
**

**Figure S5. Digital photograph (top left) and SEM image of the DAT membrane.** Scale bar, 100 μm.

**
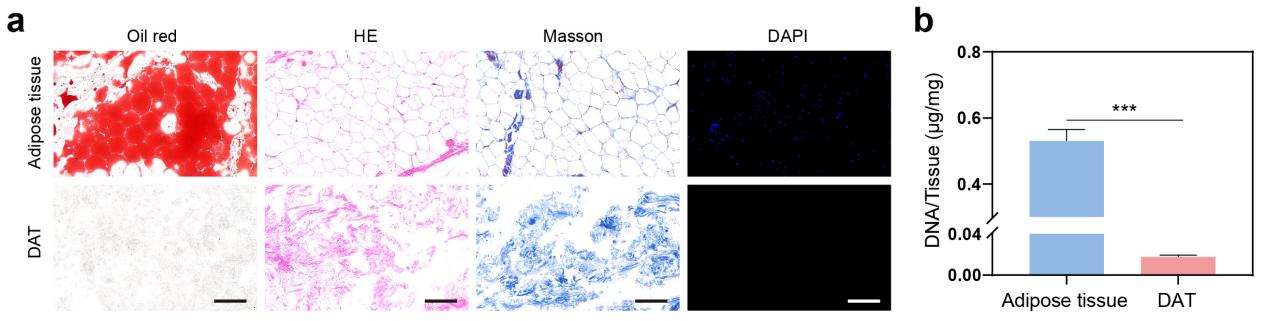
**

**Figure S6. Histological section examinations and DNA content assays of adipose tissue and DAT.** a) HE, Masson's Trichrome, Oil Red O, and DAPI staining images of the tissue samples. b) DNA content quantification in the tissue samples (n = 3, mean ± s.d.). n represents the number of biologically independent samples. Scale bar, 200 µm. (***P < 0.001).


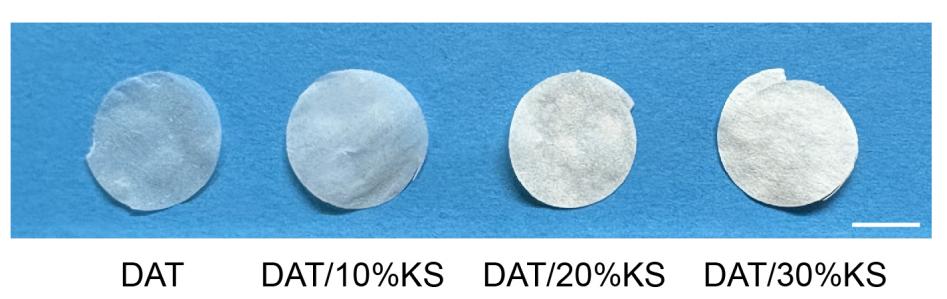


**Figure S7. Gross observation of the DAT/KS membranes with various KNN-SSD weight ratios (w/w, 10%, 20% and 30%).** Scale bar, 1 cm.

**
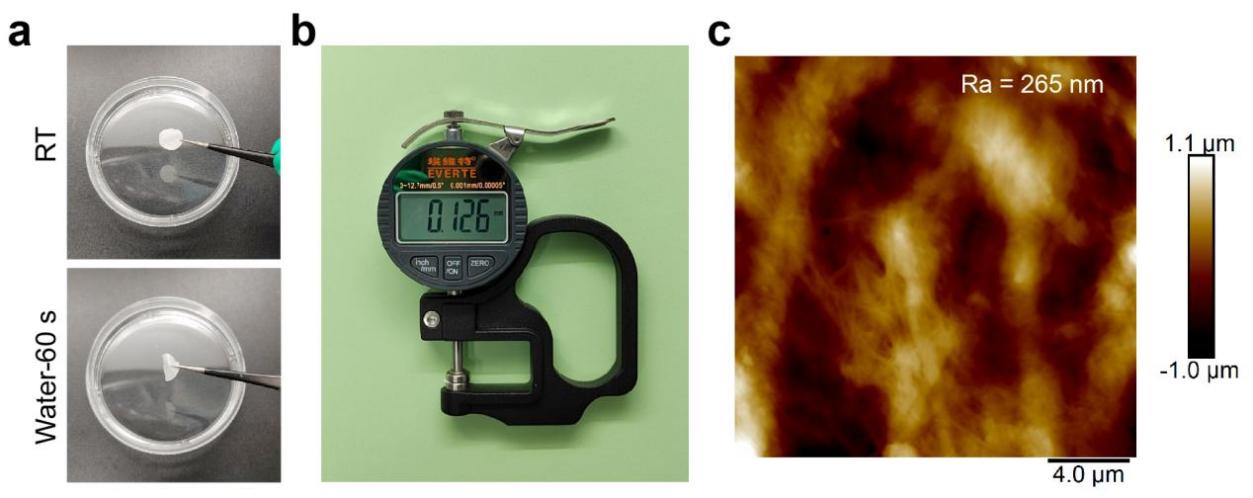
**

**Figure S8. Characterization of the DAT/KS membrane.** a) The changes of the DAT/KS membrane before and after exposure to water for 60s. b) The thickness of the DAT/KS membrane. c) AFM image of the DAT/KS membrane.


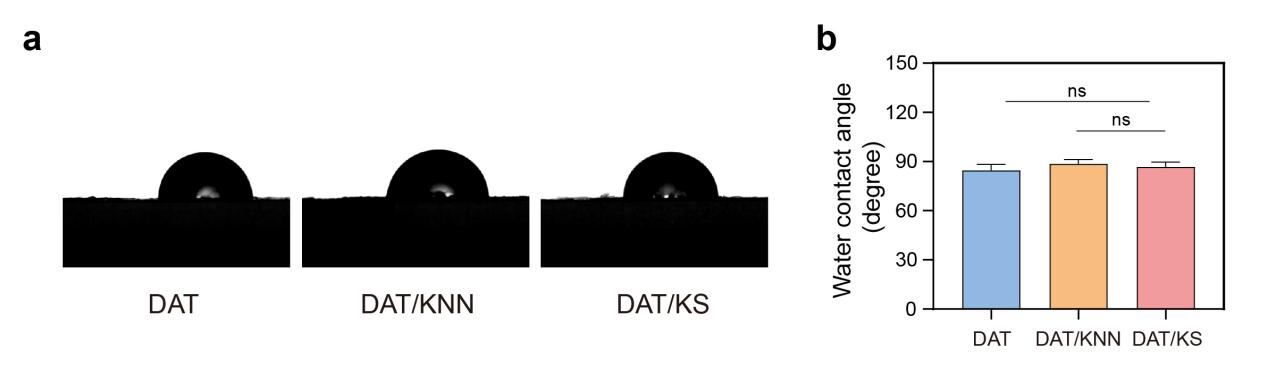


**Figure S9. Hydrophilicity analysis of the piezoelectric membranes.** a) The water contact angles of the DAT, DAT/KNN and DAT/KS membranes. b) Quantitative analysis of the water contact angle. (n = 3, mean ± s.d.). n represents the number of biologically independent samples.


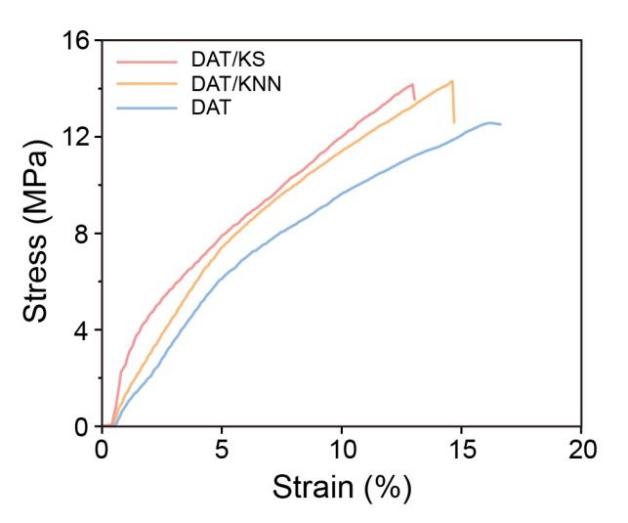


**Figure S10. Stress-strain curves of the DAT, DAT/KNN and DAT/KS membranes.**

**
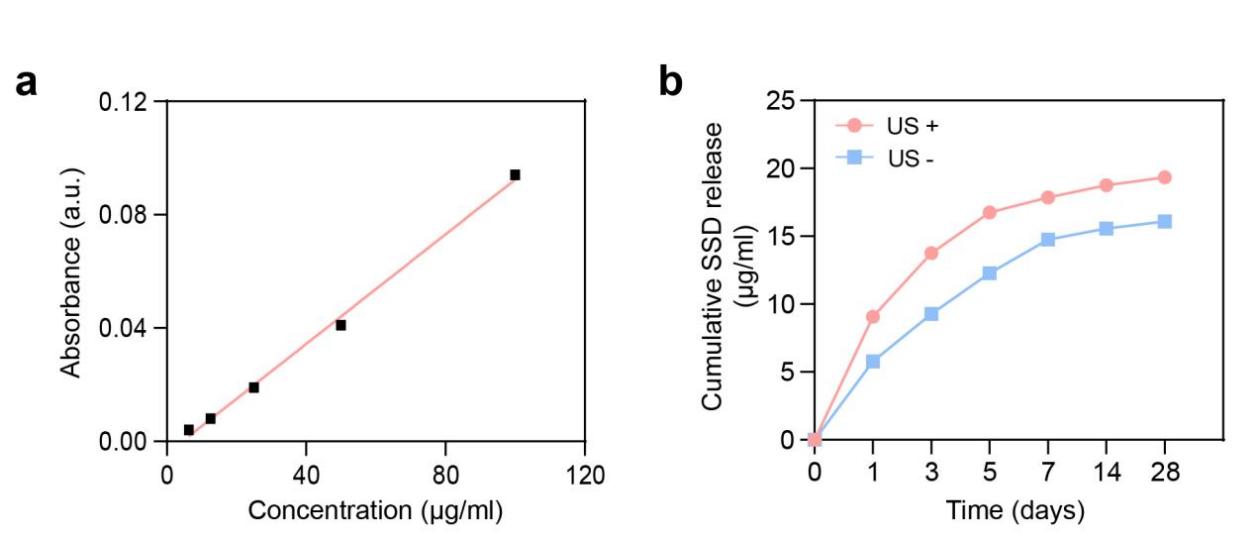
**

**Figure S11. The determination of SSD release from the DAT/KS membranes.** a) The standard curves of SSD concentrations. b) The sustained release profile of SSD from the DAT/KS membrane.

**
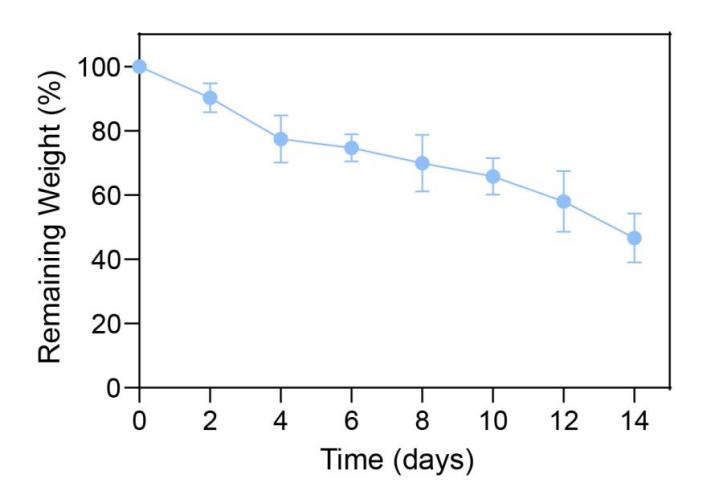
**

**Figure S12. Remaining weight curve of the DAT/KS membranes under Rapid Degradation condition (n = 3, mean ± s.d.).**

**
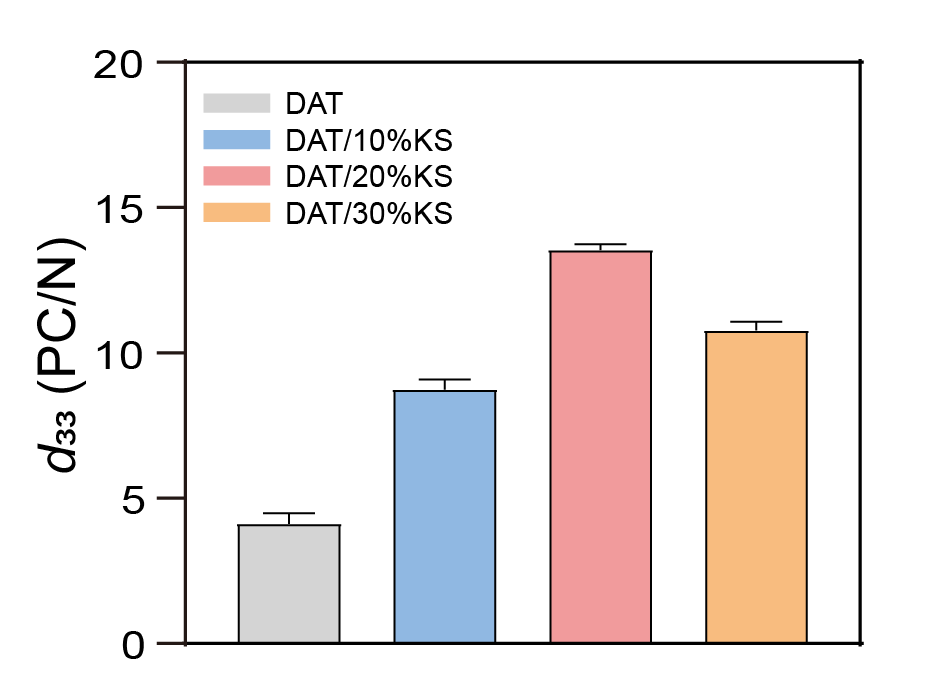
**

**Figure S13. Measurement of the piezoelectric constants *d*_33_ of the DAT/KS membrane with varying KNN-SSD mass ratios (10%, 20%, 30%) (n = 3, mean ± s.d.).**

**
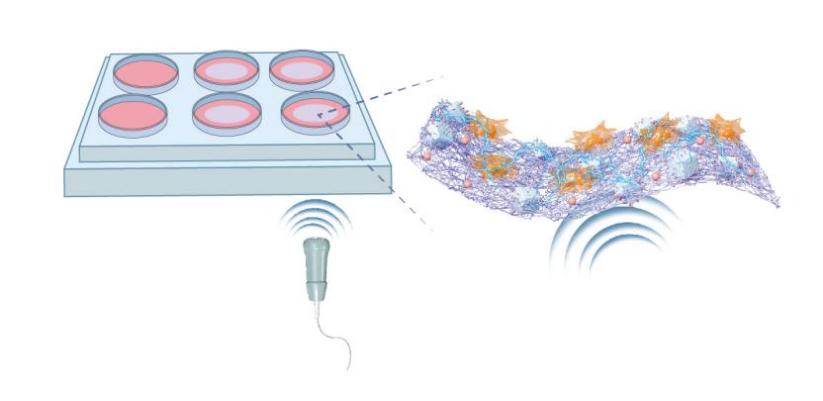
**

**Figure S14. Schematic diagram of BMSCs co-cultured with piezoelectric composite membranes and subjected to US treatment.**


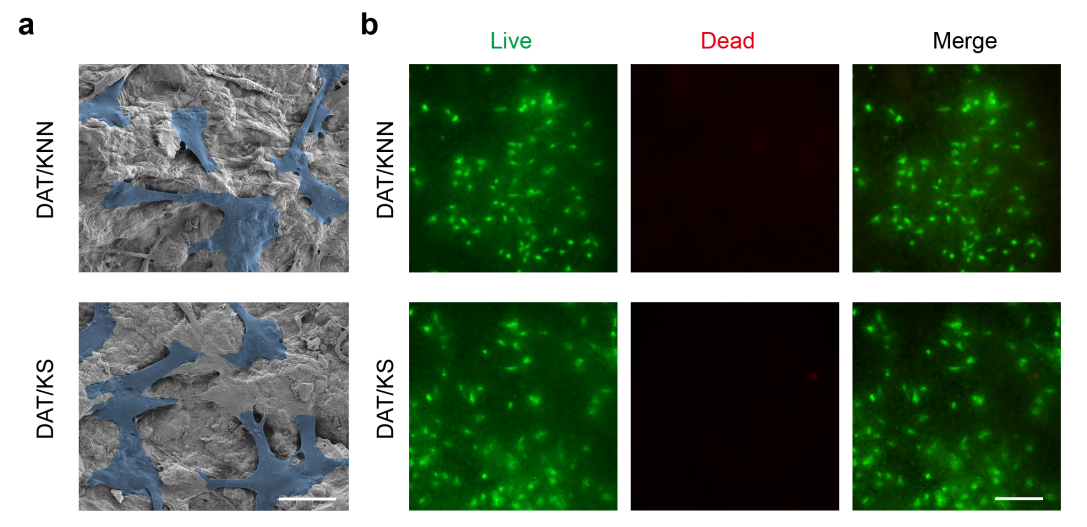


**Figure S15. The growth of BMSCs on the DAT/KNN and DAT/KS membranes.** a) SEM images of BMSCs cultured on the DAT/KNN and DAT/KS membranes after 48h. b) Live/dead staining of BMSCs cultured on the DAT/KNN and DAT/KS membranes after 48h. Scale bar, 20 μm (a), 400 μm (b).


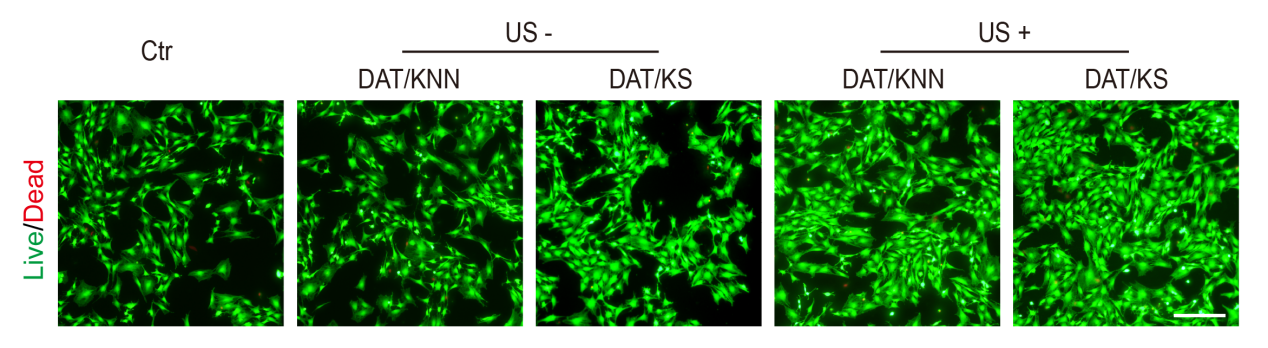


**Figure S16. Live/dead staining of BMSCs co-cultured with the DAT/KNN and DAT/KS membranes with or without ultrasound treatment after 48h.** Scale bar, 400 μm.


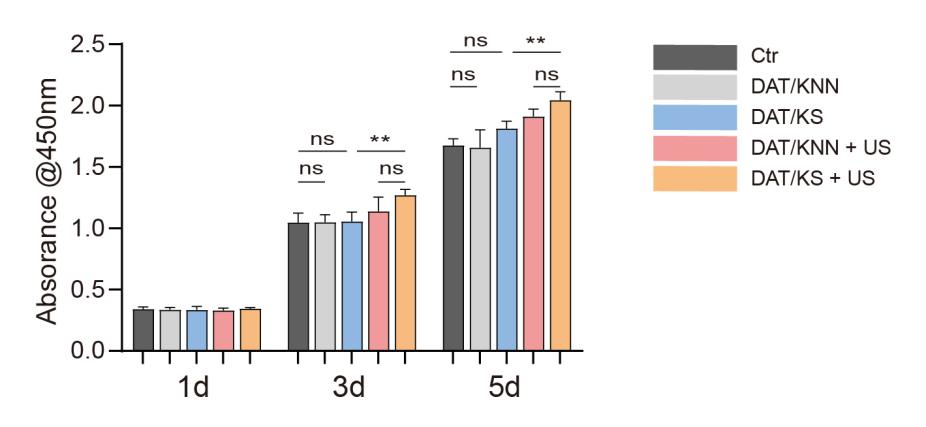


**Figure S17. CCK-8 experiments of BMSCs co-cultured with the DAT/KNN and DAT/KS membranes with or without ultrasound treatment after 1, 3 and 5 days (n = 3, mean ± s.d.).** n represents the number of biologically independent samples. ( **P < 0.01).


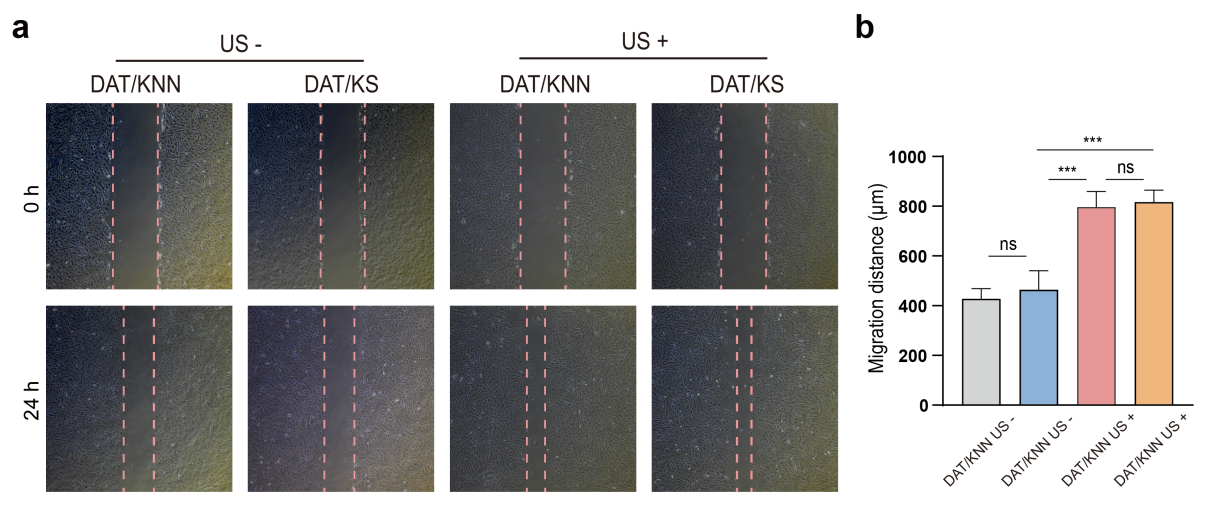


**Figure S18. Wound scratch assay (a) and semi-quantitative analysis of migration distance (b) among different groups (n = 3, mean ± s.d.).** n represents the number of biologically independent samples. ( ***P < 0.001).


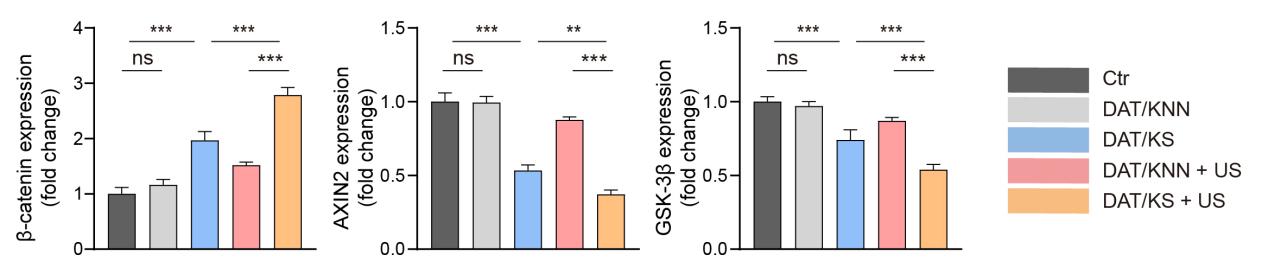


**Figure S19. Semi-quantitative analysis of β-catenin, AXIN2, and GSK-3β protein expression levels from Western blot results.** The expression of the proteins mentioned above was normalized to that of GAPDH (n = 3, mean ± s.d.). n represents the number of biologically independent samples. (**P < 0.01, *** P < 0.001).


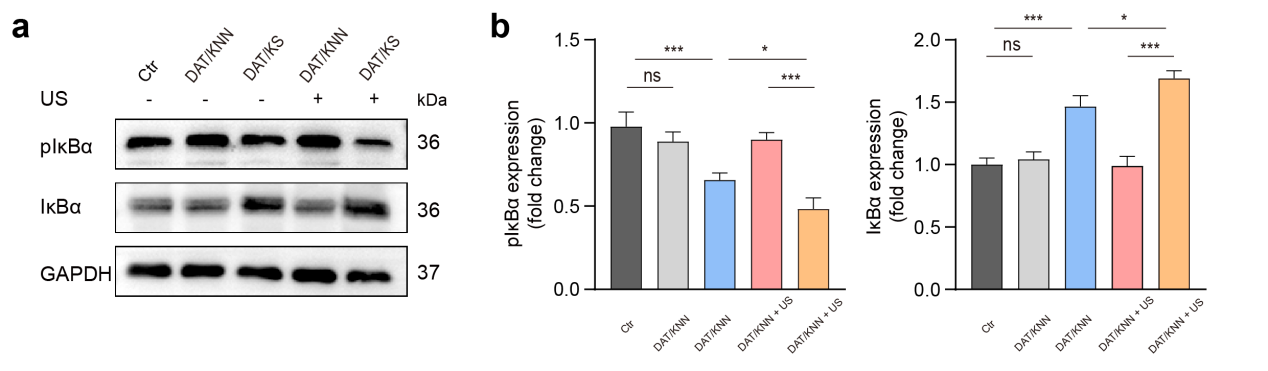


**Figure S20. The phosphorylation and degradation of IκBα are prevented by the DAT/KS membrane under the ultrasound treatment.** a) Western blot analysis of IκBα and pIκBα expressions in BMMs. b) Semi-quantitative analysis of IκBα and pIκBα protein expression level from Western blot results (n = 3, mean ± s.d.). n represents the number of biologically independent samples. (*P < 0.05, *** P < 0.001).


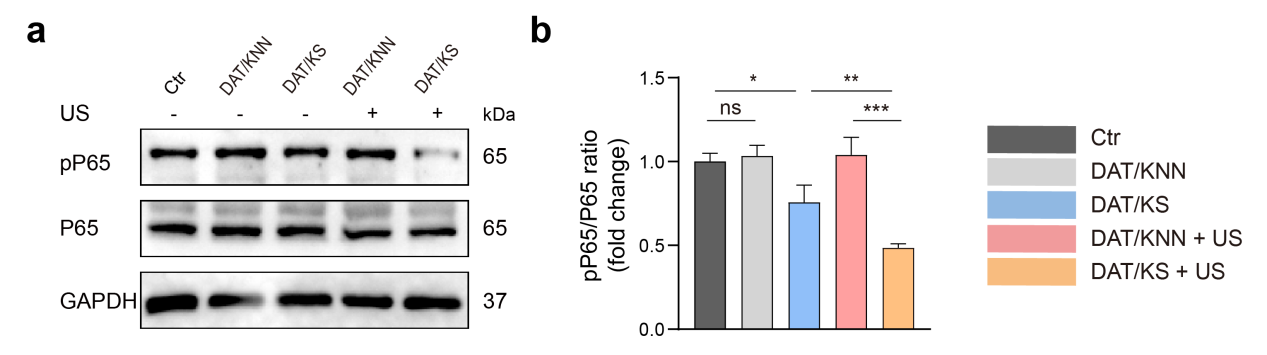


**Figure S21. The phosphorylation of P65 is inhibited by the DAT/KS membrane under the ultrasound treatment.** a) Western blot analysis of P65 and pP65 expressions in BMMs. b) Semi-quantitative analysis of pP65 protein expression level from Western blot results. The expression of pP65 proteins was normalized to that of P65 (n = 3, mean ± s.d.). n represents the number of biologically independent samples. (*P < 0.05, **P < 0.01, *** P < 0.001).

**
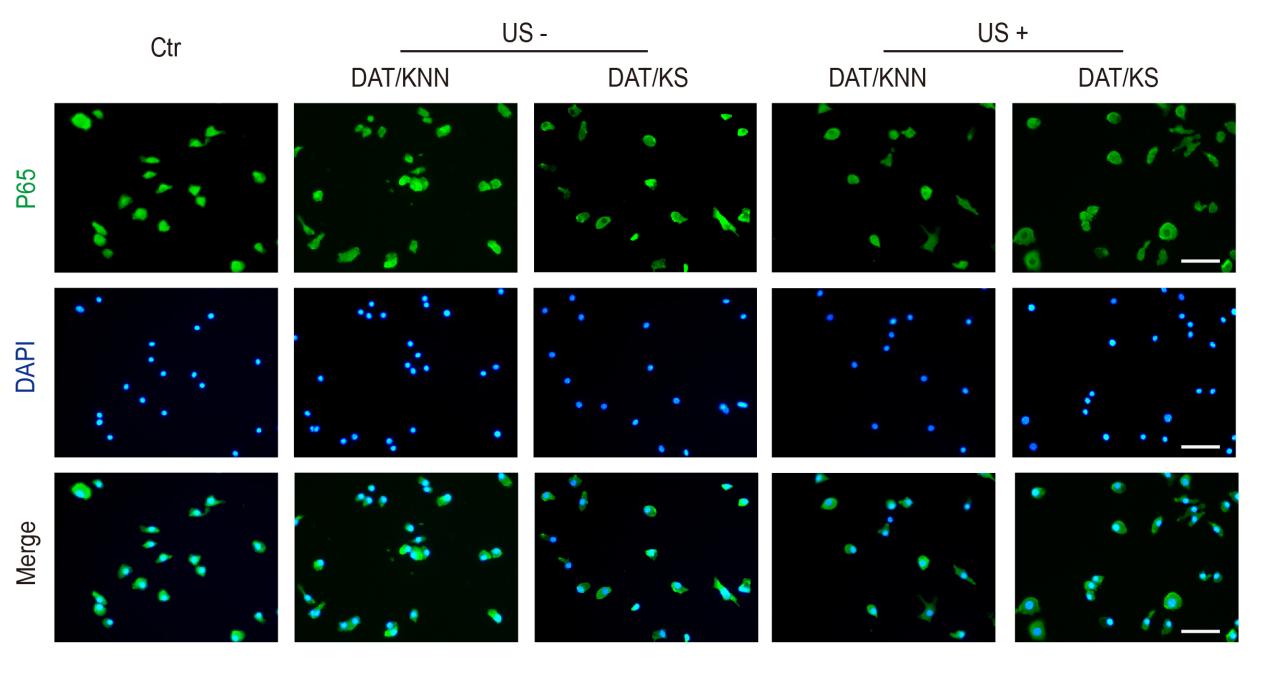
**

**Figure S22. Representative images of immunofluorescence staining displaying the localization of P65 in different experimental groups.** Scale bar, 100 μm.


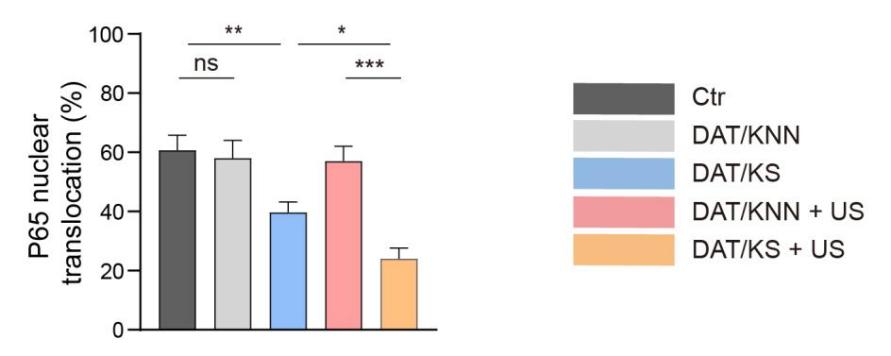


**Figure S23. Percentage of cells showing P65 nuclear translocation, calculated from three different fields (n = 3, mean ± s.d.).** n represents the number of biologically independent samples. (*P < 0.05, **P < 0.01, *** P < 0.001).


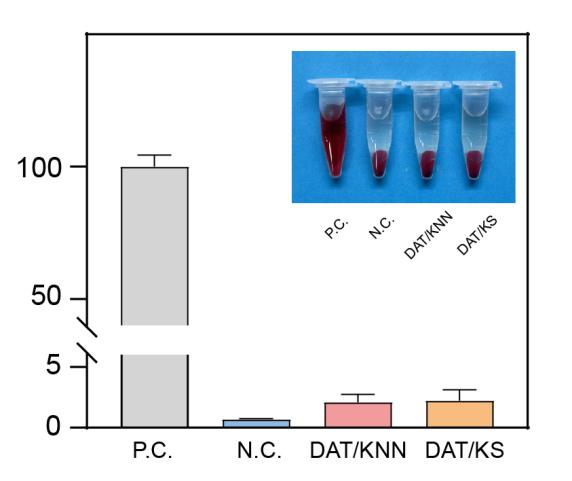


**Figure S24. Hemolysis test of the DAT/KNN and DAT/KS membranes.** Red blood dipersed in deionized water was set as the positive control group (P.C.), and 0.9 % saline to disperse the set as the negative control group (N.C.), (n = 3, mean ± s.d.). n represents the number of biologically independent samples. (*P < 0.05, **P < 0.01, *** P < 0.001).


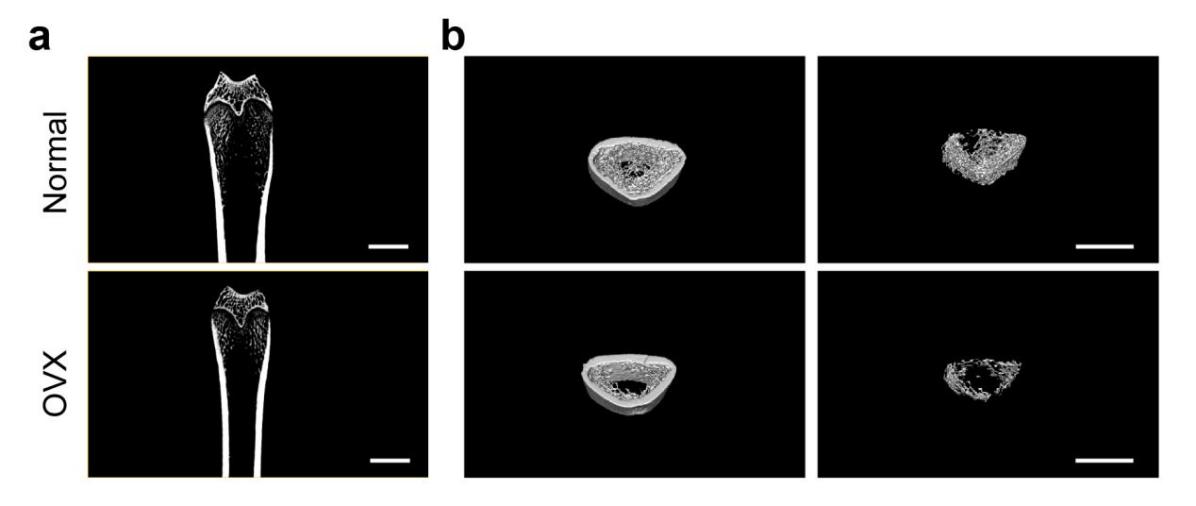


**Figure S25. A comparison of distal femur bone mass in OVX and normal rats.** MicroCT analysis of the distal femur in coronal (a) and cross-sectional (b) positions. Scale bar, 400 mm (a,b).


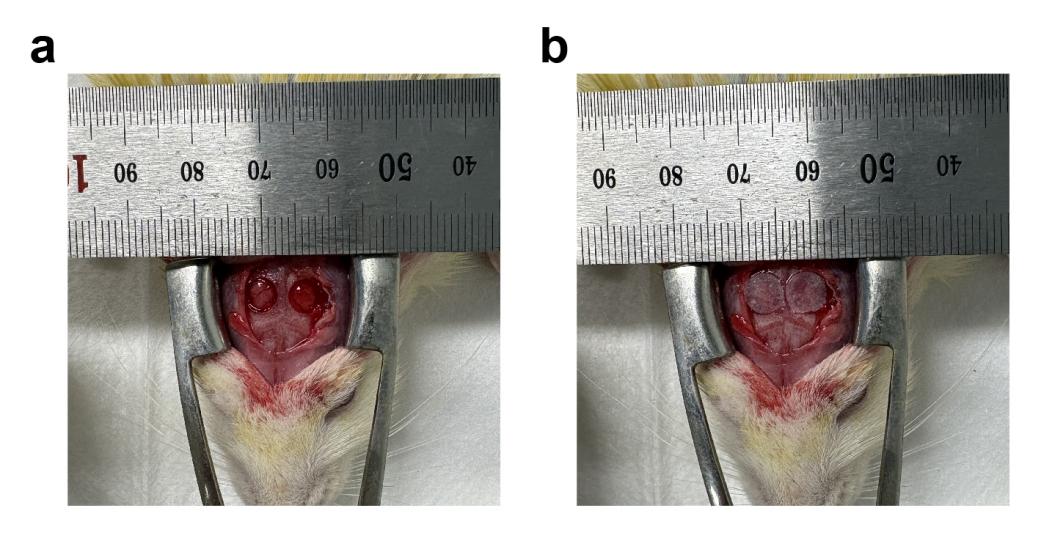


**Figure S26. Digital photographs of the cranial bone defect model (a) and the DAT/KS membranes implantation (b) in the cranial bone defect area.**


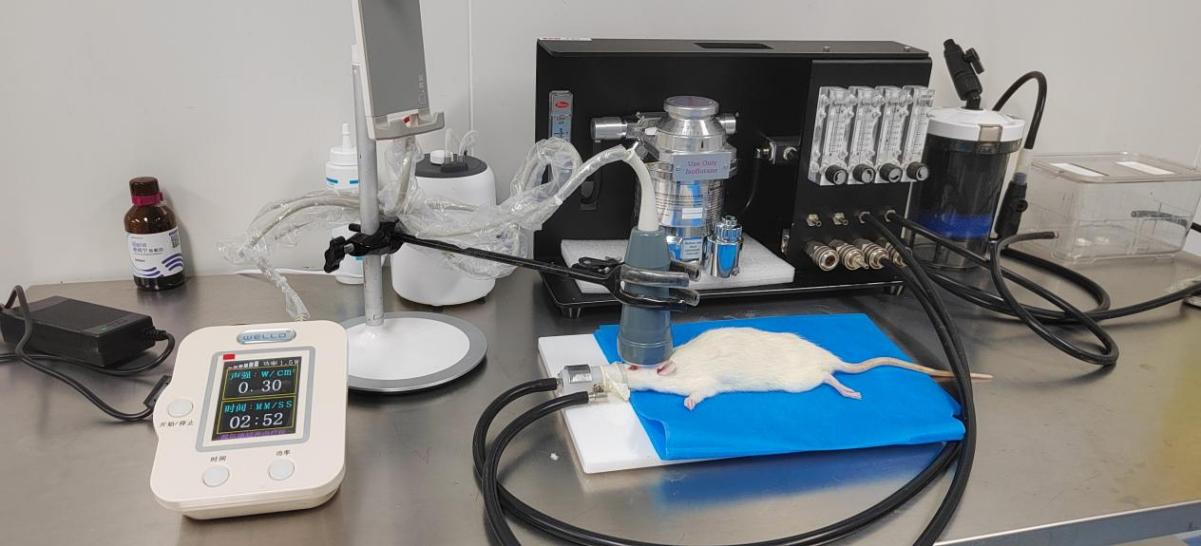


**Figure S27. Digital photograph of the OVX rat undergoing ultrasound treatment in the cranial bone defect area.**


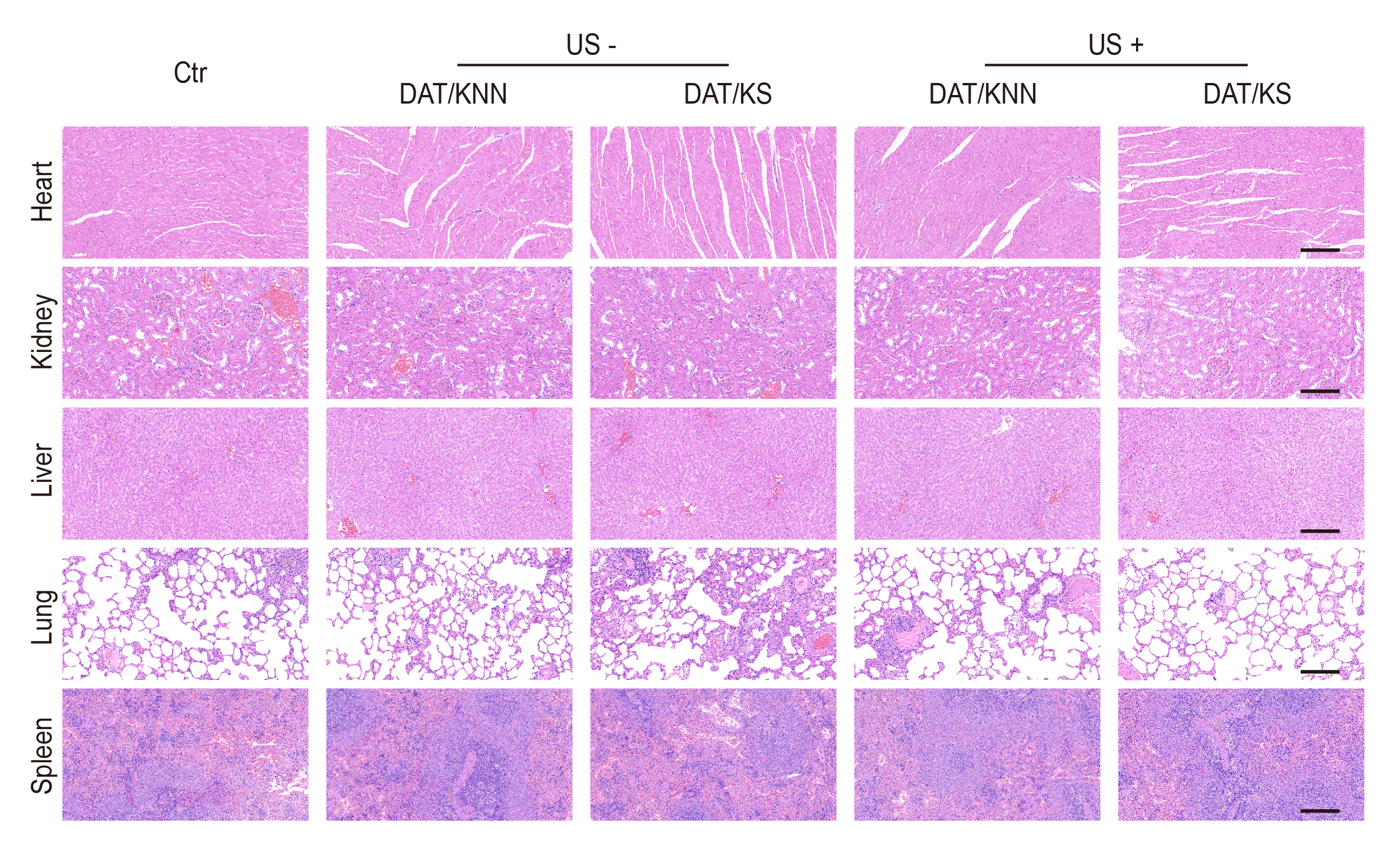


**Figure S28. In vivo safety of the piezoelectric composite membranes in SD rats.** HE staining of histological sections from major organs, including the heart, kidneys, liver, lungs, and spleen. scale bar, 200 µm.

**Table S1 Primers used for qRT-PCR of osteogenic genes (Rat)**

| **Gene** | **Forward primers (5’ to 3’)** | **Reverse primers (5’ to 3’)** |
| --- | --- | --- |
| *Alpl* | CAACCTGACTGACCCTTCCC | CGATGGCCTCATCCATCTCC |
| *Bglap* | TCAACAATGGACTTGGAGCCC | GCAACACATGCCCTAAACGG |
| *Col1a1* | ATCAGCTGGAGTTTCCGTGC | GGACCCATTGGACCTGAAGC |
| *Runx2* | GCGGTGCAAACTTTCTCCAG | TGCAGCCTTAAATGACTCGG |
| *Gapdh* | CCGCATCTTCTTGTGCAGTG | CGATACGGCCAAATCCGTTC |

**Table S2 Primers used for qRT-PCR of osteoclastogenic genes (Mouse)**

| **Gene** | **Forward primers (5’ to 3’)** | **Reverse primers (5’ to 3’)** |
| --- | --- | --- |
| *Acp5* | TGGACCCACCGCCAAGATG | CACAGCCACAAATCTCAGGGT |
| *Fos* | CGAAGGGAACGGAATAAGATG | GCTGCCAAAATAAACTCCAG |
| *Ctsk* | CCTGTTGGGCTTTCAGCTCT | CCGTTCTGCTGCACGTATTG |
| *Mmp9* | TGGTCTTCCCCAAAGACCTG | CACAGCGTGGTGTTCGAATG |
| *Gapdh* | GACACTGAGCAAGAGAGGCCCTA | TGGGATGGAAATTGTGAGGGA |
